# Refining the TCGA Framework: The Pivotal Role of Targeted Next-Generation Sequencing in Resolving Diagnostic Ambiguities in Endometrial Cancer—Systematic Review

**DOI:** 10.3390/ijms27198602

**Published:** 2026-09-25

**Authors:** Natalia Gierulska, Julia Orzelska, Katarzyna Lachowska, Amelia Trzcińska, Karolina Mazur, Natalia Picheta, Julia Piekarz, Patrycja Wójcikiewicz, Iwona Puzio, Ewa Tomaszewska, Anna Kułak, Krzysztof Kułak

**Affiliations:** 1 Students’ Scientific Association at the 1st Chair and Department of Oncological Gynecology and Gynecology, Medical University of Lublin, 20-059 Lublin, Poland; 61336@umlub.edu.pl (J.O.); 61987@umlub.edu.pl (K.L.); 61951@umlub.edu.pl (A.T.); 61619@umlub.edu.pl (K.M.); 59750@umlub.edu.pl (N.P.); 59745@umlub.edu.pl (J.P.); 59848@umlub.edu.pl (P.W.); 2 Department of Animal Physiology, University of Life Sciences in Lublin, Akademicka 12, 20-950 Lublin, Poland; iwona.puzio@up.edu.pl (I.P.);; 3 Department of Diagnostic and Microsurgery of Glaucoma, Medical University of Lublin, 20-059 Lublin, Poland; 4 1st Chair and Department of Oncological Gynecology and Gynecology, Medical University of Lublin, Staszica 16 St., 20-081 Lublin, Poland

**Keywords:** next-generation sequencing, endometrial cancer, immunohistochemistry, molecular classification, TCGA, POLE, ProMisE

## Abstract

The classification of endometrial cancer based on histological examination often fails to accurately predict treatment outcomes for patients. The Cancer Genome Atlas (TCGA) has proposed a new molecular model that more accurately identifies the tumour subtype, enabling more precise treatment. However, its implementation in daily practice remains a challenge. This systematic review assesses the key role of targeted next-generation sequencing (NGS) in refining the TCGA model and resolving these diagnostic ambiguities. A systematic review was conducted using the PubMed, Scopus, and Embase databases, focusing on studies published mainly between 2019 and 2025. Original studies and clinical guidelines concerning the evaluation of targeted NGS panels, surrogate markers, and the clinical significance of molecular subtypes in endometrial cancer were analysed. Based on the PRISMA guidelines, 11 relevant studies were used in the review. NGS is of key importance, particularly for the detection of POLE gene mutations that cannot be identified using traditional immunohistochemistry. The studies confirm that NGS accurately classifies tumours that exhibit more than one molecular feature. Although sequencing requires more time and resources, it provides a more objective and reproducible diagnostic model. Furthermore, NGS panels currently allow patients to be divided into four prognostic groups with a 100% survival rate in the POLE subtype. Across independently reported cohorts, concordance between NGS and IHC for MMR/p53 status ranged from roughly 65% to 98% depending on the platform and gene target, and NGS-based germline testing identified Lynch syndrome in 9–10% of patients screened; targeted panels also detected homologous-recombination-repair alterations (e.g., BRCA1/2, ATM, CHEK2) relevant to PARP-inhibitor eligibility. NGS combined with immunohistochemistry enables the most accurate diagnosis of patients with endometrial cancer. This combined approach reduces diagnostic errors and also provides the basis for decisions regarding personalised treatment. The introduction of targeted sequencing as a standard clinical tool allows for greater precision in tumour classification and improves the quality of patient care.

## 1. Introduction

### 1.1. Epidemiology and Risk Factors

Endometrial cancer (EC) is the sixth most common malignancy among women worldwide [1]. The majority of cases (over 90%) are diagnosed in women over the age of 50, with the average age being 63. Moreover, approximately 4% of women with EC are under the age of 40 [2]. Its incidence varies specifically from region to region, but the highest number of cases is currently reported in North America and Western Europe—10 times more than in other continents [1,3,4]. In these regions, it is the most common cancer of the female reproductive tract. In less developed countries, risk factors are less prevalent, but mortality from this condition is proportionally higher [3]. In Europe, endometrial carcinoma is the most common gynaecological cancer, with an estimated 121,578 new cases and 29,638 deaths in 2018, and its incidence has been rising with the ageing and increasing obesity of the population [5]. Currently, the primary risk factors are hyperestrogenism and obesity [6]. Other risk factors can be categorised based on their association with oestrogen levels. The first group encompasses early menarche, late menopause, tamoxifen therapy, and infertility—including lack of ovulation and polycystic ovary syndrome. The second group, independent of hormonal status, includes hypertension, diabetes, age over 50, thyroid disease, a family history of endometrial cancer, and Lynch syndrome. Conversely, long-term use of combined oral contraceptives has been shown to exert a protective effect, significantly reducing EC risk [7].

### 1.2. Survival and Prognosis

The EUROCARE-5 study reported a 5-year relative survival of 76% for European women diagnosed between 2000 and 2007, ranging from 72.9% in Eastern Europe to 83.2% in Northern Europe [5]. The majority of EC cases are diagnosed at stage I, when the cancer is not yet very advanced. The 5-year survival rate is more than 95%—the same as in stage II. However, the 5-year survival rate decreases when there is regional spread or distant disease [2,8]. When diagnosed at stage III or IV, the 5-year survival rate is only 60% [8].

### 1.3. Clinical Presentation and Diagnosis

The cardinal symptom of EC, and frequently the initial clinical presentation, is abnormal genital tract bleeding, reported by both perimenopausal and menopausal women. However, this is not a pathognomonic symptom and very often can also mean other conditions. Sometimes the symptoms of this cancer may not directly indicate problems with the reproductive organs, such as pain, abdominal bloating, and constipation or diarrhoea. Therefore, the current diagnosis of endometrial cancer should develop all the time [4].

#### Lynch Syndrome and Mismatch Repair Screening

Approximately 3% of all endometrial carcinomas, and about 10% of mismatch repair deficient (MMRd) or microsatellite-unstable endometrial carcinomas, are causally related to germline mutations in one of the MMR genes MLH1, PMS2, MSH2, and MSH6 [5]. Endometrial cancer is the most common extracolonic tumour and a sentinel cancer in women with Lynch syndrome (LS), and approximately half of the women with LS present with endometrial cancer as their initial malignancy [9]. The cumulative incidence of endometrial cancer at 70 years of age differs according to the gene affected, reaching 34% for MLH1, 51% for MSH2, 49% for MSH6, and 24% for PMS2 mutation carriers [5].

For this reason, testing of MMR status or microsatellite instability is recommended in all endometrial carcinomas irrespective of histological subtype and irrespective of patient age, both to identify patients at increased risk of LS and for diagnostic, prognostic, and predictive purposes [5]. Immunohistochemistry for MMR proteins is the preferred approach, as it is widely available, cost-effective, and additionally indicates which gene or protein is altered; molecular microsatellite instability testing is an alternative, but is more laborious, requires non-neoplastic tissue, and does not identify the affected gene [5].

Importantly, a deficient MMR result in the tumour is not in itself diagnostic of LS, since MMRd and microsatellite instability are frequently caused by sporadic events such as bi-allelic somatic mutations or promoter hypermethylation. Both approaches therefore require analysis of *MLH1* promoter methylation status in cases showing loss of MLH1/PMS2 expression, and referral for genetic counselling is recommended in the absence of hypermethylation [5]. The definitive diagnosis of LS-associated endometrial cancer requires NGS-based detection of germline mutations in MMR genes [9]. The relevance of this stepwise approach to NGS-based molecular classification is examined in Section 2.

### 1.4. Treatment Options

The standard surgical treatment of endometrial cancer is total hysterectomy with bilateral salpingo-oophorectomy, with minimally invasive surgery being the preferred approach, including in patients with high-risk disease [5]. Sentinel lymph node biopsy is an acceptable alternative to systematic lymphadenectomy for lymph node staging in stage I and II disease and can be omitted in cases without myometrial invasion [5]. In selected pre-menopausal patients under 45 years of age with low-grade endometrioid carcinoma and myometrial invasion below 50%, ovarian preservation may be considered, whereas fertility-sparing treatment with progestins may be offered to patients with atypical hyperplasia or grade 1 endometrioid carcinoma without myometrial invasion [5].

Adjuvant treatment is guided by the assigned prognostic risk group. No adjuvant treatment is recommended in low-risk disease; vaginal brachytherapy is recommended in the intermediate-risk group to reduce vaginal recurrence; and external beam radiotherapy with concurrent and adjuvant chemotherapy, or sequential chemotherapy and radiotherapy, is recommended in high-risk disease. In advanced or recurrent disease, hormonal therapy is the preferred front-line systemic treatment for low-grade tumours without rapid progression, while the combination of carboplatin and paclitaxel is the standard chemotherapy regimen [5].

Molecular classification is increasingly used to refine these decisions rather than merely to describe prognosis. In the molecular analysis of the PORTEC-3 trial, patients with p53abn tumours derived a significant benefit from combined chemoradiotherapy compared with radiotherapy alone, with a 5-year recurrence-free survival of 58.6% versus 36.2%, whereas patients with POLEmut tumours had an excellent outcome in both treatment arms, supporting consideration of de-escalation in this subgroup [10]. No benefit from added chemotherapy was observed in MMRd tumours [10]. Accordingly, current guidelines recommend that omission of adjuvant treatment should be considered in stage I–II disease classified as low-risk on the basis of a pathogenic *POLE* mutation [5].

Immune checkpoint inhibition represents a further subtype-directed option: pembrolizumab has been approved for MSI-high/MMRd solid tumours that have progressed on conventional therapy, and the combination of pembrolizumab and lenvatinib has been approved for second-line treatment of microsatellite-stable endometrial carcinoma. The predictive value of MMR status for immune checkpoint inhibitor therapy is one of the four reasons for which MMR/MSI testing is considered relevant in all endometrial carcinomas [5].

Finally, the p53abn subgroup has been shown to exhibit molecular features of homologous recombination deficiency (HRD). Germline *BRCA*-associated endometrial carcinomas with loss of heterozygosity of the wild-type allele fall almost exclusively within the TP53-mutated TCGA subgroup (91.7%) and show a copy-number high profile, in contrast to tumours without such loss, which are distributed across all four molecular subgroups [11]. This provides a rationale for evaluating poly (ADP-ribose) polymerase (PARP) inhibitors in this population, and it has been noted that patients with p53abn tumours could benefit from the addition of PARP inhibitors to chemoradiotherapy [10]. It should be emphasised, however, that tumours arising in germline *BRCA* carriers are not homologous recombination deficient by default and may therefore not respond to treatments targeting this repair defect, which is why loss of heterozygosity status should be taken into account when assessing PARP inhibitor sensitivity [11]. PARP inhibitors are not currently part of the standard management of endometrial cancer, and their role remains under investigation.

### 1.5. Molecular Classification of EC

Historically, EC was dichotomised into two basic clinical types, differing in their microscopic appearance and disease course. Type I consisted of oestrogen-dependent tumours—diagnosed more frequently, this tended to have a low degree of malignancy and was associated with a more favourable prognosis. Type II, on the other hand, was unrelated to the effects of oestrogen, and occurred less frequently, but was characterised by a higher degree of malignancy, worse prognosis, and higher mortality. A new characterisation of the tumour was proposed in 2013 by The Cancer Genome Atlas (TCGA) on the basis of integrated genomic, transcriptomic, and proteomic analyses, which allowed the delineation of four molecular subtypes of EC [12]. All are listed in Table 1.

#### 1.5.1. The TCGA Classification

The basic molecular classification on which this entire review is based stems from a 2013 TCGA analysis encompassing 373 cases of endometrial cancer. The study utilised integrated genomic, transcriptomic, and proteomic sequencing to define four subtypes with different prognoses, including: POLE-ultramutated (≈7% of cases), hypermutated with microsatellite instability (≈28%), low copy number (≈39%), and high copy number (≈26%) [12].

#### 1.5.2. Surrogate Classifiers: ProMisE and TransPORTEC

Although the TCGA analysis provided a biologically informative framework, the methods used were impractical for routine diagnostic work: they relied on fresh-frozen tumour material requiring special handling and on costly and complex methodologies that were considered too expensive for routine clinical use [14,15,16]. Two research groups therefore developed pragmatic assays that identify four analogous, but not identical, molecular subgroups using techniques that can be applied to formalin-fixed paraffin-embedded material in most pathology laboratories [14,16,17].

The Proactive Molecular Risk Classifier for Endometrial Cancer (ProMisE) is a decision tree of binary parameters based on the presence or absence of a protein or a mutation. Tumours are assessed sequentially: first by immunohistochemistry for mismatch repair proteins, then by sequencing of the *POLE* exonuclease domain, and finally by p53 immunohistochemistry, yielding the subgroups MMR-D, *POLE* EDM, p53 wild-type, and p53 abnormal [14,15]. MMR testing was deliberately placed first because this information prompts early referral to hereditary cancer programmes for Lynch syndrome testing [14]. ProMisE has completed the discovery, confirmation, and validation stages of the Institute of Medicine framework for the development of omics-based tests, the last in a population-based cohort of 452 consecutive endometrial carcinomas [17].

Because these surrogate assays work on FFPE material, they can also be applied to diagnostic specimens obtained before surgery, which the original TCGA methodology could not. Agreement between molecular classification performed on diagnostic biopsy or curettage specimens and on the subsequent hysterectomy specimen was near perfect in two independent series, with a kappa statistic of 0.86 (*n* = 57) and 0.88 (*n* = 156), far exceeding the agreement reported for histotype (kappa 0.44) and grade (weighted kappa 0.70) between the same specimen types [14,17]. Molecular subtype is therefore available from the time of first diagnosis and can inform the timing and extent of surgery [14,17].

The TransPORTEC consortium, related to the PORTEC-3 trial, developed a comparable approach, combining p53 immunohistochemistry, PCR-based microsatellite instability testing, and Sanger sequencing of the *POLE* exonuclease domain, initially in a series of 116 high-risk endometrial cancers and subsequently in 834 early-stage endometrioid carcinomas from the PORTEC-1 and PORTEC-2 trials [16,18]. In the latter analysis, molecular classification was feasible in more than 96% of cases and, when integrated with established clinicopathological factors, produced a stronger risk model than clinicopathological assessment alone [18].

It should be emphasised that these classifiers are not equivalent to the original TCGA classification but represent clinically feasible approximations of it, and that the two systems differ in their technical implementation: ProMisE uses ultra-deep targeted sequencing of *POLE* exons 9–14, whereas the TransPORTEC studies used Sanger sequencing of exons 9 and 13 [14,16]. Their principal differences are summarised in Table 2.

### 1.6. Next-Generation Sequencing

Next-generation sequencing (NGS) is the technology that has enabled this classification. It is a high-throughput method that allows for the parallel sequencing of millions of DNA fragments, enabling the analysis of targeted gene panels, entire exomes, or entire genomes. The specific application of NGS depends on the particular clinical issue and the sequencing chemistry used. Sequencing chemistries include processes such as reversible terminator chemistry, which is used to generate these data [19,20,21,22].

It can be applied to isolate and analyse different parts of the genome depending on the clinical question at hand. We can divide its applications into whole genome sequencing (WGS), targeted genome sequencing, chromatin immunoprecipitation sequencing (ChIP-seq), and RNA sequencing (RNA-Seq) [23].

WGS involves sequencing the entire genome and obtaining the most complete picture of the genome, which is important when studying complex phenotypic traits and diseases. A limitation of this approach is that WGS is generally performed at a relatively low sequencing depth, which reduces sensitivity for variants present at low allele frequency. Increasing the depth required to detect such variants reliably raises the cost, the turnaround time, and the computational burden of the analysis. Despite generating vast amounts of data that need to be processed, WGS has great clinical potential and may be useful in precision medicine [24]. In cases where a person’s illness or condition has already been diagnosed, we most often use the method of choice targeted genome sequencing. It provides both greater coverage of the regions of the genome we want to study and is more affordable [23].

ChIP-seq maps protein–DNA interactions and histone modifications rather than DNA sequence variants and is therefore a research tool rather than a method used for the molecular classification of EC in clinical practice [25].

RNA-Seq is used for gene expression analysis and for the detection of novel transcripts and isoforms and can identify diagnostic and prognostic biomarkers [26]. Its accuracy depends strongly on the quality and quantity of the RNA input, and RNA is more susceptible to degradation than DNA, which limits its applicability to routine FFPE-based diagnostics [27]. For these reasons, neither RNA-Seq nor ChIP-seq is used as a primary method for the molecular classification of EC, and the remainder of this review focuses on WGS-, targeted-NGS-, and IHC-based approaches. Since the molecular classification of endometrial cancer can be approached either by protein-based or by sequence-based methods, and since each resolves a different part of the classification, the principal features of immunohistochemistry, targeted NGS panels, and whole-genome or whole-exome sequencing are compared in Table 3.

### 1.7. Aim of the Review

The aim of this systematic review is to assess the role of targeted next-generation sequencing in refining the TCGA-based molecular classification of endometrial cancer, with particular focus on the diagnostic ambiguities that cannot be resolved by immunohistochemistry alone, namely the detection of POLE mutations and the classification of tumours displaying more than one molecular feature.

## 2. Materials and Methods

### 2.1. Study Design

This systematic review was conducted in accordance with the 2020 ‘Preferred Reporting Items for Systematic Reviews and Meta-Analyses’ (PRISMA) guidelines. It is a systematic review of the published literature on the role of targeted next-generation sequencing (NGS) in the molecular classification of endometrial cancer (EC) by The Cancer Genome Atlas (TCGA). The review protocol was registered in the International Prospective Register of Systematic Reviews (PROSPERO) under registration number CRD420261356093. The completed PRISMA 2020 checklist is provided in the Supplementary Materials.

### 2.2. Search Strategy

A search for articles on NGS use in endometrial cancer was initiated in October 2025. A comprehensive literature search was conducted in the following electronic databases: PubMed, Scopus, and Embase. The search mainly covered studies published between 2019 and 2025. However, a few older studies were included for substantive reasons. The following keywords were used as search terms: “endometrial cancer” AND “next-generation sequencing” (PubMed—364 results, Scopus—474 results, Embase—428 results), “targeted sequencing” AND “endometrial cancer” (PubMed—512 results, Scopus—304 results, Embase—465 results), “endometrium” AND “TCGA” (PubMed—57 results, Scopus—1219 results, Embase—142 results), “endometrial cancer” AND “immunohistochemistry” (PubMed—1156 results, Scopus—3544 results, Embase—2180 results), “endometrial cancer” AND “molecular classification” (PubMed—543 results, Scopus—960 results, Embase—734 results), “next-generation sequencing” AND “immunohistochemistry” AND “endometrial cancer” (PubMed—135 results, Scopus- 263 results, Embase—188 results).

### 2.3. Eligibility Criteria

We included studies that met the following criteria: original research articles, systematic reviews, clinical practice guidelines, and narrative reviews published in peer-reviewed journals; studies on the molecular classification of endometrial cancer using the TCGA framework or TCGA surrogate classifiers (including ProMisE and TransPORTEC); studies evaluating the use of targeted NGS panels, whole-exome sequencing in the context of molecular subtyping of endometrial cancer; studies comparing NGS-based approaches with methods based on immunohistochemistry (IHC) or other molecular methods in the classification of endometrial cancer. We also focused on studies that assessed the clinical utility, diagnostic accuracy, prognostic implications, and therapeutic impact of NGS-based molecular classification in endometrial cancer. All included studies had to be published in English. We excluded studies that were case reports involving fewer than three patients, unless they provided unique molecular information relevant to the objectives of the review (e.g., synchronous endometrial and ovarian cancers assessed using NGS). During the writing of the paper, editorial articles, commentaries, or letters to the editor without original data were also excluded. If articles written in a language other than English did not have a translation available, they were also excluded. We also did not use publications that had been withdrawn or retracted.

### 2.4. Study Selection

Five reviewers (N.G., J.O., K.L., A.T., K.M.) independently evaluated the titles and abstracts of the articles to identify those that met the inclusion criteria. The same five reviewers then assessed full texts of potentially relevant reports. Inconsistencies at either stage were resolved through discussion. When consensus could not be reached, six different reviewers (N.P, J.P., I.P., E.T., A.K., K.K.) were appointed to adjudicate. A total of 13,669 records were identified (PubMed *n* = 2767; Scopus *n* = 6764; Embase *n* = 4138). Of these, 13,266 were removed before screening (duplicate records, *n* = 3142; records published before 2019, *n* = 4580; records removed for other reasons, *n* = 5544—including records that, after a title review, were deemed unrelated to endometrial cancer or to molecular classification/next-generation sequencing, reflecting the broad scope of the individual search terms used). These records were removed during the initial relevance screening, which was conducted prior to the formal analysis of the titles and abstracts of the remaining records, leaving 403 records for title and abstract screening. At this stage, 322 records were excluded because the title and abstract indicated that the study did not address the molecular classification of endometrial cancer or the use of NGS, and it could therefore be rejected without full-text review. The remaining 81 reports were sought for retrieval; of these, 19 could not be obtained, leaving 62 reports assessed for eligibility at full text. This second assessment applied the complete eligibility criteria, including the type of sequencing performed, the classification system used, and the availability of comparative or outcome data—none of which can be reliably determined from an abstract alone.

Of these, 51 were excluded (no original data on NGS-based EC molecular classification, *n* = 18; insufficient methodological detail for quality assessment, *n* = 14; review not meeting inclusion criteria, *n* = 13; outcomes not relevant to review objectives, *n* = 6), leaving 11 studies included in the review, all of which are tabulated in Table 4. The full PRISMA 2020 flow diagram is presented in Figure 1. A summary of every study is provided in the Supplementary Materials (Table S1).

### 2.5. Data Extraction

The data were collected independently by five reviewers using a standard data extraction form. The extracted variables are summarised in Table 5. Any discrepancies were resolved through discussion or consultation with the sixth and seventh reviewers.

### 2.6. Quality Assessment

The methodological quality of the included studies was appraised using design-specific tools: studies on diagnostic accuracy and platform concordance (*n* = 6) were assessed using the Quality Assessment of Diagnostic Accuracy Studies-2 (QUADAS-2), whereas observational cohort studies (*n* = 4) were evaluated across the selection, comparability, and outcome domains of the Newcastle–Ottawa Scale (NOS). The single-patient case report (*n* = 1) was evaluated qualitatively. Risk of bias figures were generated using the robvis visualisation tool. The assessment was performed independently by two reviewers, with discrepancies resolved by consensus. The methodological quality and domain-level risk-of-bias judgements across the included studies are summarised in Figure 2 and Figure 3 below.

The methodological quality of the included studies was satisfactory, and most areas were assessed as having a low risk of systematic error in both assessment tools. Of the six studies assessed using QUADAS-2, patient selection, the reference standard, and the study design and duration were predominantly assessed as having a low risk of bias, reflecting generally well-designed diagnostic comparisons. The main area requiring some caution was the index test, where the use of blinding was not always explicitly reported (Guo et al., Yoshimura et al., Paratore et al., and Jamieson et al.), whilst only two studies (Dedeurwaerdere et al. and Rivera et al.) identified individual high-risk domains [9,29,30,32,33,36]. However, this in-depth analysis did not undermine the overall pattern of consistency observed across the entire evidence base. Among the four cohort studies assessed using the NOS, sample selection and outcome determination were generally well conducted, whilst comparability was the main area requiring cautious interpretation. Only one study (Alba Mota et al.) raised additional concerns. In summary, these results confirm the generally robust evidence base regarding the role of targeted next-generation sequencing (NGS) in improving TCGA-based molecular classification, whilst the limitations identified reflect the exploratory, real-world nature of the cohorts included, rather than systematic flaws in the study design [38].

### 2.7. Data Synthesis and Analysis

In writing this paper, we focused on a descriptive synthesis, given the diversity of study designs, molecular platforms, and outcome measures across the included studies. The studies were organised into several thematic areas. The first area concerned the epidemiology and risk factors of endometrial cancer, highlighting the clinical need for an improved molecular classification. The second area provided an overview of the TCGA molecular classification framework, including the four molecular subgroups and their prognostic significance. We focused on incorporating landmark studies such as the PORTEC-3 molecular classification. The third section focused on molecular diagnostic methods. It describes and compares alternative classifiers based on IHC, multiplex immunofluorescence/immunohistochemistry, PCR-based MSI tests, and NGS-based approaches. The fourth section dealt with NGS technologies and platforms. It also covers the technical principles of targeted NGS, including library preparation, sequencing chemistry, and bioinformatics processes. The fifth area assessed the clinical utility of targeted next-generation sequencing (NGS) in the molecular classification of endometrial cancer. We summarised the evidence regarding the use of NGS in resolving diagnostic uncertainties, reclassifying tumours originally classified using immunohistochemistry (IHC)-based methods, identifying co-occurring molecular alterations, and detecting Lynch syndrome. The sixth section compared NGS with other molecular methods, analysing agreement, discordance, and complementarity between NGS and IHC/PCR-based methods. The seventh and final area focused on the limitations and barriers to the implementation of NGS. It covered costs, infrastructure, bioinformatics expertise, turnaround time, and challenges related to standardisation. No forest plot analyses or meta-analyses were conducted due to the heterogeneity of the research projects and molecular platforms.

## 3. Results

In 2025, Qiuli Teng et al. published a study employing a simplified, one-step NGS panel including POLE, TP53, and microsatellite instability (MSI) to classify EC. They collected 233 formalin-fixed paraffin-embedded (FFEE) samples retrospectively from patients with EC. Data on overall survival (OS) were available for 131 patients with a median follow-up of 66 months. Genomic DNA was extracted and subsequently analysed using a single-step NGS panel, including measurements of TP53, POLE, and MSI. Comprehensive genomic analyses were performed on patients with DNA subtypes with MSI-H and CNL subtypes. The results of the study showed that the molecular typing rate among 233 patients was 8.15% for the POLE subtype, 18.88% for the MSI-H subtype, 11.59% for the CNH subtype, and 61.37% for the CNL subtype. Furthermore, the 10-year overall survival (OS) and disease-specific survival (DSS) rates were highest among patients in the POLE subtype (100%), whilst in patients with the CNH subtype, they were only 33.51% and 39.69%. Their finding indicate that this streamlined NGS-based classifier can effectively categorise EC patients into four prognostic molecular subtypes [35,39].

In a retrospective study by Guo et al. (2024), a compact NGS panel covering 46 genes was applied to 331 patients with endometrial cancer, 284 of whom had endometrioid carcinoma [28]. It showed 91.8% agreement with IHC results regarding MSI-H/MMR status. A 65.3% agreement rate was also demonstrated regarding p53/TP53 status. Furthermore, the study identified pathogenic variants in genes responsible for homologous recombination repair at the following rates: BRCA1 3.6%, BRCA2 9.7%, ATM 12.7%, and CHEK2 2.7% [28]. These alterations are of potential relevance for the evaluation of PARP inhibitors in endometrial cancer, although this approach is not currently part of standard clinical management and remains investigational [10,11,28]. A separate NGS-based ProMisE test was also validated against the original ProMisE classifier. Both were shown to have high agreement while maintaining prognostic value. This supports molecular subtyping based on NGS performed directly on biopsy material collected at the time of initial diagnosis [29].

This pattern of agreement is confirmed by a previous comparative study involving 49 tumour samples, in which NGS-based MSI detection achieved an AUC of 0.93 compared to the IHC method. At the same time, it matched and exceeded the performance of PCR and the Idylla platform, as confirmed by an AUC of 0.91 for both tests. All three molecular tests achieved 100% sensitivity and specificity when the tumour cell content reached at least 30% [30]. Consistent with the screening algorithm, NGS-based subtyping directly supports the identification of Lynch syndrome. In the cohort, 90 patients with EC were classified according to molecular subtypes. Among them, the following mutations were present with the following frequencies: POLE 12.2%, MMRd 21.1%, p53abn 6.7%, and NSMP 60%. In this group, germline MMR gene sequencing identified pathogenic variants in 9 out of 90 patients, representing 10%. Additionally, seven of them belonged to the MMRd molecular subtype, and two to the NSMP subtype. This underscores that the molecular subtype alone is not a substitute for germline testing [9].

NGS should be conducted in cases where the test outcome can directly influence therapeutic decisions and where approved targeted treatments are available for the detected molecular alterations. In addition to endometrial cancer, NGS is used in other cancers such as lung adenocarcinoma, ovarian cancers, prostate cancers, and cholangiocarcinoma. In other types of cancer, NGS may be considered in the context of clinical trials, compassionate use, or early access programmes for investigational treatments, or when standard therapeutic options have been depleted [30,39,40].

### Other Molecular Methods

The continuous development of medicine not only enables the advancement of innovative therapies using new drugs but also leads to changes in the approach to diagnosis and detection of diseases. In oncology, technological advances have enabled significant advancements, including the establishment of molecular categorisation of cancers. While the histological classification of endometrial cancer remains the foundation for evaluation and guides clinical decisions, there has been a notable rise in the significance of molecular classification. The combination of these two approaches has been shown to facilitate a more precise determination of the specific type of cancer, thereby enabling the selection of the most suitable therapeutic intervention and enhancing patient prognoses. Furthermore, it facilitates the design of clinical trials focusing on targeted therapies. It is especially important in complicated situations, cases with poor prognosis, or recurrences of endometrial cancer [10,13,41]. IHC is a well-known antigen-based technique for detecting the expression of specific proteins in tumour tissues. It allows characterisation of cell phenotype, including markers of proliferation, apoptosis, differentiation, or specific tumour receptors. This method has been shown to detect only a singular marker on a tissue slice, thereby limiting the comprehensive analysis of the tumour microenvironment (TME). This phenomenon complicates the analysis of intercellular interactions and the determination of co-expressions of markers within a single cell. It also should be noted that staining may exhibit overlap or interfere visually, especially when multiple chromogenes are employed. Furthermore, conventional IHC may prove inadequate in the evaluation of intricate cellular systems, such as immune infiltration. Consequently, new techniques are being developed to augment IHC and enhance its capabilities [40,42,43]. One of these techniques is multiplex immunofluorescence (mIF)—it allows the detection of multiple protein markers within a single tissue sample by employing various fluorochromes at the same time. Furthermore, spatial analysis of cells and their interactions in the TME is a potential avenue for further research. This method is of particular significance in the field of cancer immunotherapy, as it facilitates the identification of diverse immune cell populations. It assists in the identification of prognostic and predictive biomarkers, contributing to the assessment of immune responses and the monitoring of immunotherapy efficacy [43,44]. When executed with precision, mIF is known to exhibit high sensitivity and specificity; however, fluorochromes are susceptible to photobleaching, which is a form of photodegradation [43,45]. The validation and optimisation of marker panels, advanced data analysis, and specialised software represent some of the challenges associated with introducing this method into clinical practice. At this stage, mIF results must be correlated with IHC results [46]. A similar technique that also allows precise determination of a tumour’s immune profile is multiplex immunohistochemistry (mIHC). This method of marker detection is based on an enzymatic colour reaction and also allows for the detection of multiple protein markers; however, the number of protein markers detected is smaller than in mIF. As with the traditional IHC method, problems with colour overlap can emerge in this procedure as well, reducing its overall sensitivity. The implementation of mIHC in routine diagnostics is facilitated by its lower hardware requirements, staining stability, and simplified image analysis [13,44,45].

Individualising the treatment process for patients requires the development of an algorithm that often combines several techniques so that the molecular classification is as precise as possible [31,40]. A study by Rios-Doria et al. integrated next-generation sequencing (NGS) and IHC data to assess the consistency and completeness of molecular classification, taking into account their clinical utility.

A total of 1834 patients were included in the study, with the following distribution of endometrial cancer subtypes: NSMP 31.3% (575/1834), MMRd 23.3% (428/1834), p53abn 40% (734/1834), and POLEmut 5.3% (97/1834) [31]. A high concordance was observed between IHC for p53/MMR and NGS data, with a percentage exceeding 95%. NGS was imperative for the identification of POLEmut, which are not discernible through IHC. Furthermore, 6% of cases exhibited characteristics indicative of more than one molecular group, as evidenced by the presence of both POLEmut and p53abn [31]. In such cases, POLEmut dominated prognostically, in accordance with the findings in previous studies. Consequently, POLE testing should be considered a mandatory component of endometrial cancer classification, which requires the implementation of NGS technologies [31,41]. The integrated IHC and NGS algorithm has been demonstrated to be both effective and clinically feasible. IHC is sufficient for the evaluation of MMRd and p53abn, while NGS is necessary for the detection of POLEmut. NSMP is classified by exclusion. The implementation of sequencing in a limited number of cases (e.g., IHC-negative for MMR and p53) has been demonstrated to enhance cost efficiency and diagnostic efficiency while sustaining optimal classification accuracy [31]. The new diagnostic pathway proposed by the authors has the potential to be implemented in routine clinical practice. The reclassification pattern observed in this cohort is illustrated in Figure 4.

## 4. Discussion

The diagnostic landscape of EC is currently undergoing a paradigm shift, transitioning from purely morphological assessment to an integrated molecular–pathological approach. While IHC remains a fundamental tool, its role is increasingly being supplemented by NGS. Key markers used in IHC include p53, mismatch repair markers mutS homologue (MSH-6), and postmeiotic segregation increased (PMS-2) [5,46]. The most prevalent aberrant IHC patterns involve the loss of MLH-1/PMS-2 or MSH-2/MSH-6 complexes, as well as isolated losses of MSH-6 or PMS-2 [13]. However, while performing IHC, it is possible to encounter difficulties during the interpretation of results; thus, IHC should be performed by an experienced pathologist [47]. The main advantage of NGS is its ability to resolve the issue of multiple classifications. In cases where a patient has mutations in both the POLE and TP53 genes, the status of the POLE gene mutation is of decisive prognostic importance. Without the use of NGS, such a patient might be incorrectly classified into the p53abn group, which has the worst prognosis. This could lead to unnecessary and harmful intensification of treatment [32,48]. Recent evidence suggests that NGS offers a more objective and reproducible framework compared to IHC [32]. While IHC requires substantial tissue sections to evaluate multiple protein expressions, which can rapidly deplete limited diagnostic material, NGS utilises DNA amplification—enabling the analysis of limited tumour samples [32]. This approach holds particular relevance in the context of EC, where many patients experience discomfort with the procedure of collecting material for testing [49,50]. Despite the fact that both methods demonstrate high diagnostic yield, NGS provides a superior advantage by enabling the simultaneous analysis of hundreds of genes within a single workflow [47]. A retrospective study performed by Rios-Doria et al. [31] had the aim of evaluating how the integration of NGS with IHC could enhance the molecular classification of endometrial carcinoma within the four molecular subgroups originally defined by TCGA, implemented as an integrated IHC/NGS algorithm. The study involved a cohort of 1834 patients and mostly used a pre-operative biopsy or hysterectomy specimen, which was then used as a DNA sample for IHC and NGS. Among the cases studied, the majority were ECs of the CNH molecular subtype (40%), followed by those of CNL subtype (32%), those with high MSI (23%), and those of the POLE subtype (5%). Significantly, histological and genomic variability was observed across all molecular subtypes. The study demonstrated that molecular classification had prognostic significance in both early and advanced stages of the disease. The research proves that a significant proportion of cases would be misclassified or remain unclassified if the TCGA framework were based solely on MMR/p53 IHC without POLE sequencing [31]. This was proven by integrating NGS in the process of tumour-normal sequencing data. More specifically, the most commonly misclassified area by IHC was in the sequencing of the TP53 gene. Discrepancies between phenotypic protein expression and genotypic mutations were further elucidated in a study of 63 high-grade endometrioid EC cases. The cases included patients who underwent a hysterectomy with bilateral salpingo-oophorectomy in the period 2018–2020, due to their diagnosis of high-grade endometrioid EC. Out of 20 cases with TP53 mutations, 2 showed a mismatch between IHC and genetic testing NGS. Case EC-50 (serous carcinoma): IHC showed strong abnormal p53 expression, but initial NGS showed no mutation. A repeat test on a better-quality tumour sample revealed a pathogenic TP53 mutation (Gly266Arg), confirming the IHC result was correct. Case EC-58 (low-grade endometrioid carcinoma): IHC showed low p53 expression (considered normal), but NGS identified a pathogenic mutation (Gly245Asp), confirmed by Sanger sequencing. These findings underscore that while IHC is a reliable screening tool, molecular confirmation is indispensable for definitive subtyping in borderline or high-grade cases [36]. The clinical utility of NGS was also confirmed by Yoshimura et al. [32], who demonstrated in their study that NGS-based classification significantly outperforms the ProMisE classifier (based on IHC) in terms of prognostic accuracy with regard to overall survival. This study included 200 patients with EC who underwent surgery—including hysterectomy. Classification based on NGS identified the CN-L subtype as the most common (104/200, 52.0%), followed by MSI-H (38/200, 19.0%), POLE (33/200, 16.5%), and CN-H (25/200, 12.5%). OS differed significantly across the four subtypes based on NGS, compared to the ProMisE classification, where OS was not as variable. Additional mutations were misidentified for some POLE subtypes outside known hotspots, with 18.2% (6 out of 33) showing concurrent MSI-H. IHC revealed a wild-type p53 pattern for 12 (48%) CN-H cases. Furthermore, no significant difference in prognosis was observed depending on p53 status in the CN-H subtype [32]. Although NSMP is usually the most common subtype, NGS technology enables its precise classification by identifying mutations in genes such as PIK3CA, PTEN, and KRAS. This level of detail paves the way for targeted therapies and offers a significant advantage over the traditional IHC method [32,33]. Similarly, a study by Paratore et al. [33] demonstrated that NGS not only improves risk stratification but also enables the identification of therapeutic targets for personalised therapies, such as PARP inhibitors or immunotherapy, which are often overlooked by traditional methods. This study included 85 patients. Multigene profiling was performed using an NGS panel. The following mutations were detected in the patients: 5.9% POLE, 25.8% microsatellite instability with mismatch repair deficiency (MMRd/MSI), 11.8% p53abn/TP53mut, and 56.5% NSMP. Good agreement (97.6%) was demonstrated in the analysis based on MSI NGS and MMR IHC results, whilst the concordance rate for p53 IHC and TP53 mutations was 92.3% The analysis found that molecular subtypes varied significantly according to grade, FIGO stage, and lymphatic invasion (LVSI). This confirms the effectiveness of NGS and its reliability in distinguishing between both MSI and TP53-mutated tumours. This study also examined mutations in the PIK3CA, PTEN, KRAS, ERBB2, and ESR1 genes, where potential for treatment was noted [33,44]. The two studies are compared in Table 6. These two studies were selected because each directly compared molecular classification based on next-generation sequencing (NGS) with an established reference method (IHC/ProMisE) in a specific cohort of patients with endometrial cancer. At the same time, complementary aspects of the clinical utility of NGS were presented, such as prognostic accuracy and the identification of actionable therapeutic targets, including potential targets for PARP inhibitors and immunotherapy [32,33]. Furthermore, Figure 5 shows a direct visual comparison of the distribution of molecular subtypes described in key genomic studies.

Despite its advantages, the implementation of NGS is unfortunately associated with certain limitations, primarily the high cost of equipment, the need for specialist bioinformatics infrastructure, and longer waiting times for results compared to the IHC method. These challenges can be mitigated by centralising testing in reference centres, such as in a ‘hub-and-spoke’ model, which allows for cost optimisation. Furthermore, the use of targeted gene panels instead of whole-exome sequencing speeds up analysis and simplifies the interpretation of clinical results [35,39]. A significant advantage that outweighs all the above-mentioned disadvantages is the possibility of designing appropriate panels specific to a given tumour. Thanks to increasing automation of the process and reduced operator involvement, implementing this procedure for all solid tumours is more cost-effective and can be used as a standard of care for identifying gene variants [30].

Beyond primary diagnosis, NGS serves as a critical tool for differential diagnosis in complex oncological scenarios. In a case report published by Nadejda Valtcheva et al. [37], a patient was presented with a rare instance of concurrent endometrioid adenocarcinoma of the corpus uteri and the left ovary, accompanied by metastasis of endometrioid adenocarcinoma to the lungs four years after surgical intervention, brachytherapy, and tibolone therapy. In order to distinguish between the tumours as either independent neoplasms or metastases, a targeted next-generation sequencing (NGS) approach was employed, focusing on 409 genes associated with cancer [37].

The analysis revealed that the mutation profiles of both tumours exhibited 67% identity, thereby indicating their shared origin. Conversely, the lung tumour exhibited a distinct profile compared to the endometrial and ovarian cancers [37].

Distinguishing between a primary tumour and a metastasis in cases where both are present in the endometrium and ovary is imperative. In such cases, the concurrence of two cancers, endometrial cancer and ovarian cancer, is associated with a more favourable prognosis compared to endometrial cancer that has metastasised to the ovary. This distinction is clinically vital, as synchronous primaries carry a significantly more favourable prognosis than FIGO stage IV metastatic disease, fundamentally altering the therapeutic strategy [51,52,53,54,55].

Furthermore, study by Alba Mota et al. also employed targeted sequencing to discern discrepancies in mutations between primary tumours and metastases [38]. Moreover, it illustrates that NGS can reconstruct the evolutionary trajectories of tumours, providing insights into subclonal architecture and the genomic drivers of recurrence and aggressiveness [38].

### 4.1. Limitations and Barriers to Clinical Implementation of NGS

Unfortunately, NGS itself has limitations and barriers that must also be kept in mind when performing it. These include the technical and methodological aspects of NGS. The most important thing during testing is to maintain the purity of the sample, which is particularly difficult in the case of FFPE samples [34]. The integrity of the sample prior to sectioning is critical. From the moment of collection to the time of fixation, the time to fixation and storage conditions greatly affect the nucleic acids in tumour tissue. Particularly in the case of FFPE samples, the process of fixing samples in formalin can induce cross-linking of nucleic acids and proteins, leading to their modification and fragmentation [56]. An additional difficulty that arises when using the NGS method is the uneven sequence coverage within the genome, which manifests itself in difficulties in accurately detecting complex structural changes, repeats, trinucleotide sequence expansions, polyploidy, mosaicism, or variants with high sequence homology. NGS typically uses approximately 100–500 bp, which is considered a short read, and therefore difficulties may arise in detecting structural variants, making the entire method costly. Errors may also occur during reading, e.g., data analysis, gene amplification, or sequencing itself. Disturbances are particularly evident in certain areas of genes with a high content of guanine and cytosine fragments, sequence repeatability, or homopolymers. This demonstrates that despite the high efficiency of the entire NGS process, there are still inherent limitations to the entire process [34]. In addition to the low availability of this test due to its specialised nature and complexity, its turnaround time is between 5 and 10 days, whereas with other methods, the average time is around 1–2 days. NGS data analysis generates vast amounts of data that require appropriate infrastructure for backup storage, versioning, compliance, and auditing. Furthermore, there is a minimum level of cancer cells that must be present in the tissue, which is as high as 30%. Unfortunately, due to its high price, it is not always the first choice. However, if laboratories already have sequencing capabilities, and in the case of previously sequenced tumour types, the cost of performing this procedure decreases [30,57].

### 4.2. Emerging Long-Read Sequencing Technologies and Future Directions

In addition to the short-read NGS methods discussed in this review, third-generation sequencing platforms utilising long reads are increasingly being investigated in oncology. These mainly include technologies from Oxford Nanopore Technologies (ONT) and HiFi/SMRT sequencing from Pacific Biosciences (PacBio) [58]. Unlike NGS, these platforms generate reads ranging in length from a few kilobases to entire chromosome arms, improving the resolution of features such as complex structural rearrangements, repetitive elements, homopolymers, and GC-rich regions [58]. In ‘proof-of-concept’ oncology cohorts, long-read sequencing enabled the identification of clinically significant structural variants and gene fusions that had been missed by short reads. Furthermore, long-read sequencing has enabled the phasing of haplotypes of co-occurring variants [58,59]. Another potentially important advantage specific to the classification of endometrial cancer is that nanopore sequencing allows the detection of cytosine methylation directly from native DNA, without the need for bisulphite conversion [58]. MMR-deficient endometrial cancers are predominantly caused by hypermethylation of the MLH1 promoter. Unfortunately, this is not detected by the targeted NGS panels discussed here. This requires a separate bisulphite-based assay or a methylation-specific test. This procedure is based on long reads and could, in theory, combine several factors at once, including the identification of mutations, the detection of structural variants, and the determination of MLH1 methylation status, all within a single test. This would likely help to distinguish sporadic MMR deficiency from Lynch-syndrome-associated deficiency and to resolve cases involving co-occurring mutations, such as POLE and p53abn. Comparable procedures have already been prospectively evaluated in the case of central nervous system tumours. The results of these studies demonstrated concordance with standard-of-care tests of over 89% and a turnaround time of just 72 h from sample collection to report issuance [60,61]. Despite this potential, the routine use of this method in clinical practice for endometrial cancer is not currently feasible and would require significant additional validation studies. The first problem is that the vast majority of diagnostic samples for endometrial cancer are fixed in formalin and embedded in paraffin. Furthermore, long-read platforms perform optimally with intact, high-molecular-weight DNA. Consequently, the fragmentation of DNA derived from FFPE remains a major practical obstacle for long-read-based processes. Dedicated protocols for FFPE library preparation offer hope, as they are beginning to overcome this limitation. Another limitation is the cost of reagents and equipment. Unfortunately, these are not yet standardised or widely available outside specialist laboratories or those affiliated with research centres, in contrast to commercial short-read sequencing kits. The most significant limitation is that no study to date has specifically validated long-read sequencing for the classification of endometrial cancer based on TCGA, ProMisE, or NGS-integrated approaches [58,59,60,61].

## 5. Conclusions

In conclusion, the molecular classification of EC has fundamentally redefined the diagnostic and prognostic standards in gynaecological oncology. NGS plays a pivotal role by identifying difficult-to-detect mutations such as TP53 or POLE genes and assessing microsatellite instability. Currently, the synergistic application of NGS and IHC represents the most reliable and accurate diagnostic algorithm in EC diagnosis. Their simultaneous use allows for the complementation of the inherent limitations of each individual method. IHC allows for the accurate detection of p53 and MMR mutations, but the use of NGS is necessary to detect POLE mutations. In addition, NGS enables the analysis of complex clinical problems, allowing for more accurate treatment planning and better assessment of disease progression. The integration of these technologies facilitates personalised treatment planning, enhances the precision of risk stratification, and ultimately improves clinical outcomes for patients with endometrial cancer. Currently, the IHC technique is widely available and sufficient for identifying the basic MMRd and p53abn subtypes within the existing classification. However, it generally does not allow for the resolution of diagnostic uncertainties in cases with multiple classifiers, nor does it enable the detection of isolated POLE gene mutations. For this reason, targeted NGS provides a crucial level of clarity that redefines the prognosis. The use of this method in diagnosis helps to prevent erroneous prognostic assumptions as well as the overuse of toxic anticancer drugs. This, in turn, enables the provision of truly personalised and precision-based care and treatment pathways. In summary, the studies presented in this review point to a consistent pattern. The concordance between NGS and IHC results in the detection of MMRd/p53abn mutations is generally high. However, this agreement varies depending on the platform and the target gene. In turn, the POLE gene mutation status—which determines some of the most pronounced prognostic differences, including the most favourable survival outcomes among the four TCGA subtypes—cannot be reliably assessed at all using IHC. This is a key, consistent argument in favour of supplementing IHC with targeted NGS testing, rather than treating these two methods as interchangeable.

## Figures and Tables

**Figure 1:**
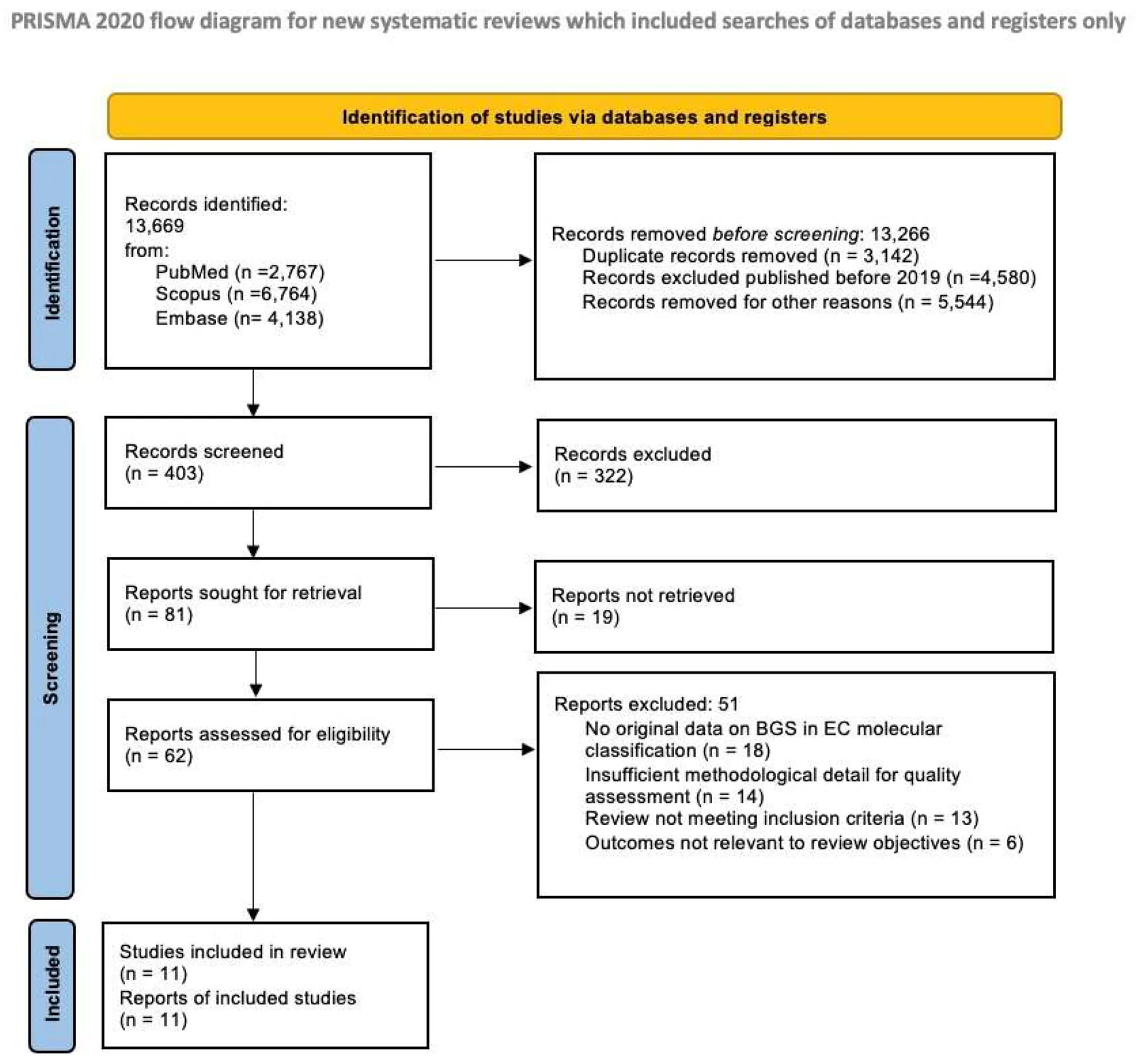
Preferred Reporting Items for Systematic Reviews and Meta-Analyses (PRISMA) flow diagram of study identification, inclusion, and exclusion.

**Figure 2:**
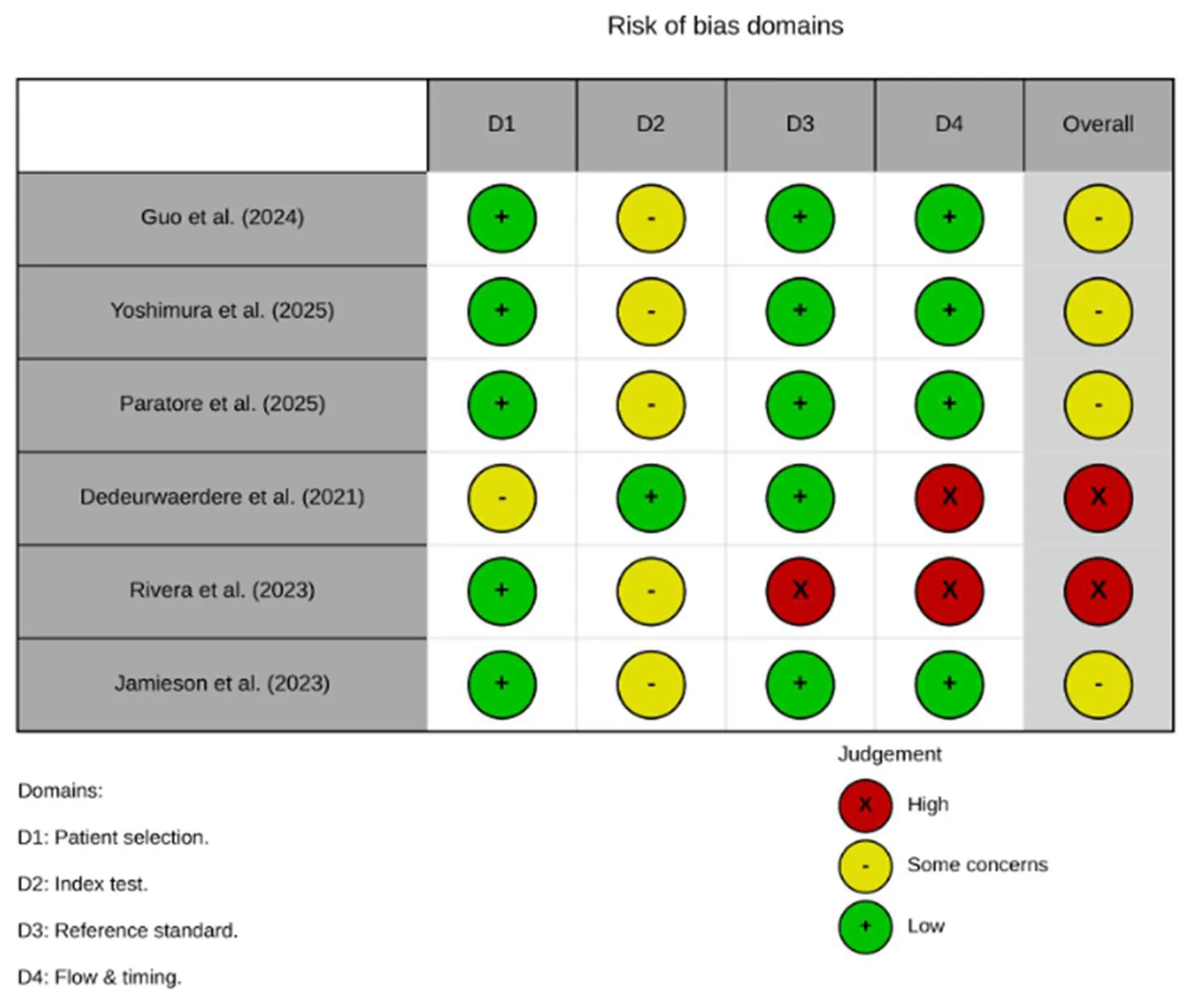
Methodological quality summary for diagnostic accuracy and concordance studies evaluated via the QUADAS-2 tool [28,29,30,32,33,36].

**Figure 3:**
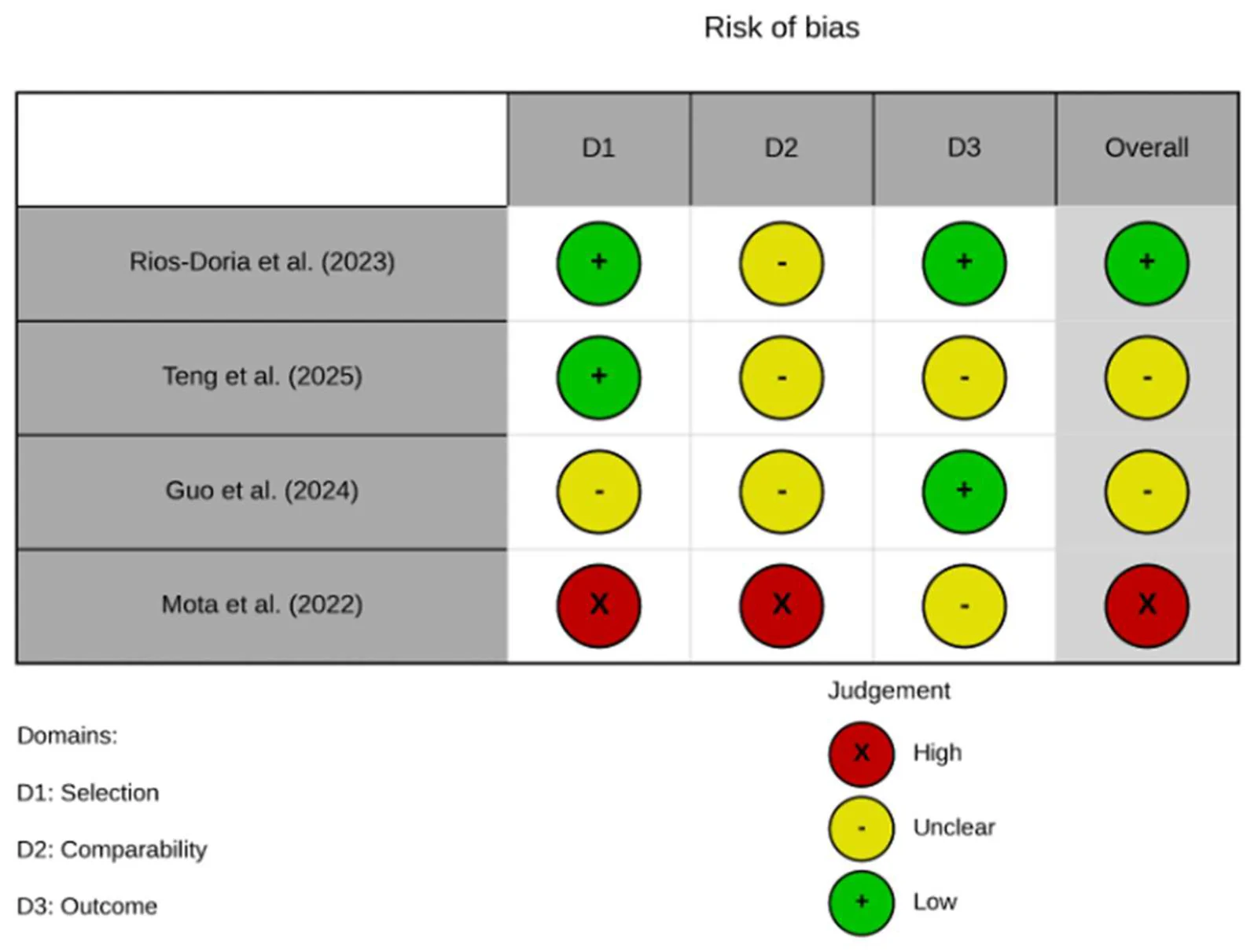
Risk of bias evaluation for observational cohort studies across Newcastle–Ottawa domains (D1: Selection, D2: Comparability, D3: Outcome). Valtcheva et al. (2017) [37] represents an illustrative single-case report (*n* = 1) evaluating tumour clonality in synchronous primary lesions. Due to the absence of a defined cohort, control group, or consecutive sampling series, it inherently falls outside the structural scope of multi-domain quantitative scoring tools (QUADAS-2 and NOS) and was consequently appraised qualitatively [9,31,35,38].

**Figure 4:**
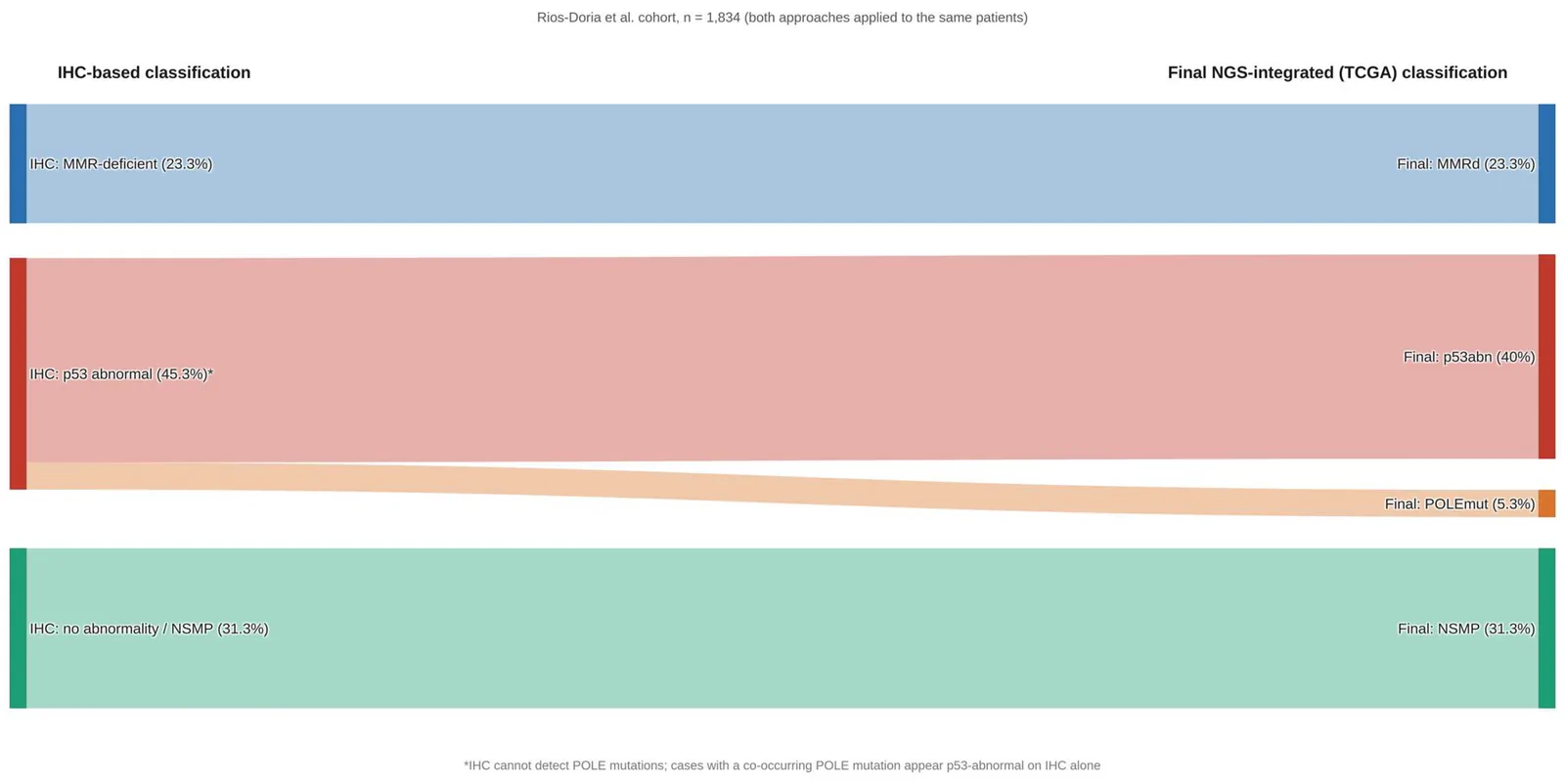
Correlation between the determination of molecular subtypes of endometrial cancer using IHC and an NGS-integrated method (*n* = 1834) [31].

**Figure 5:**
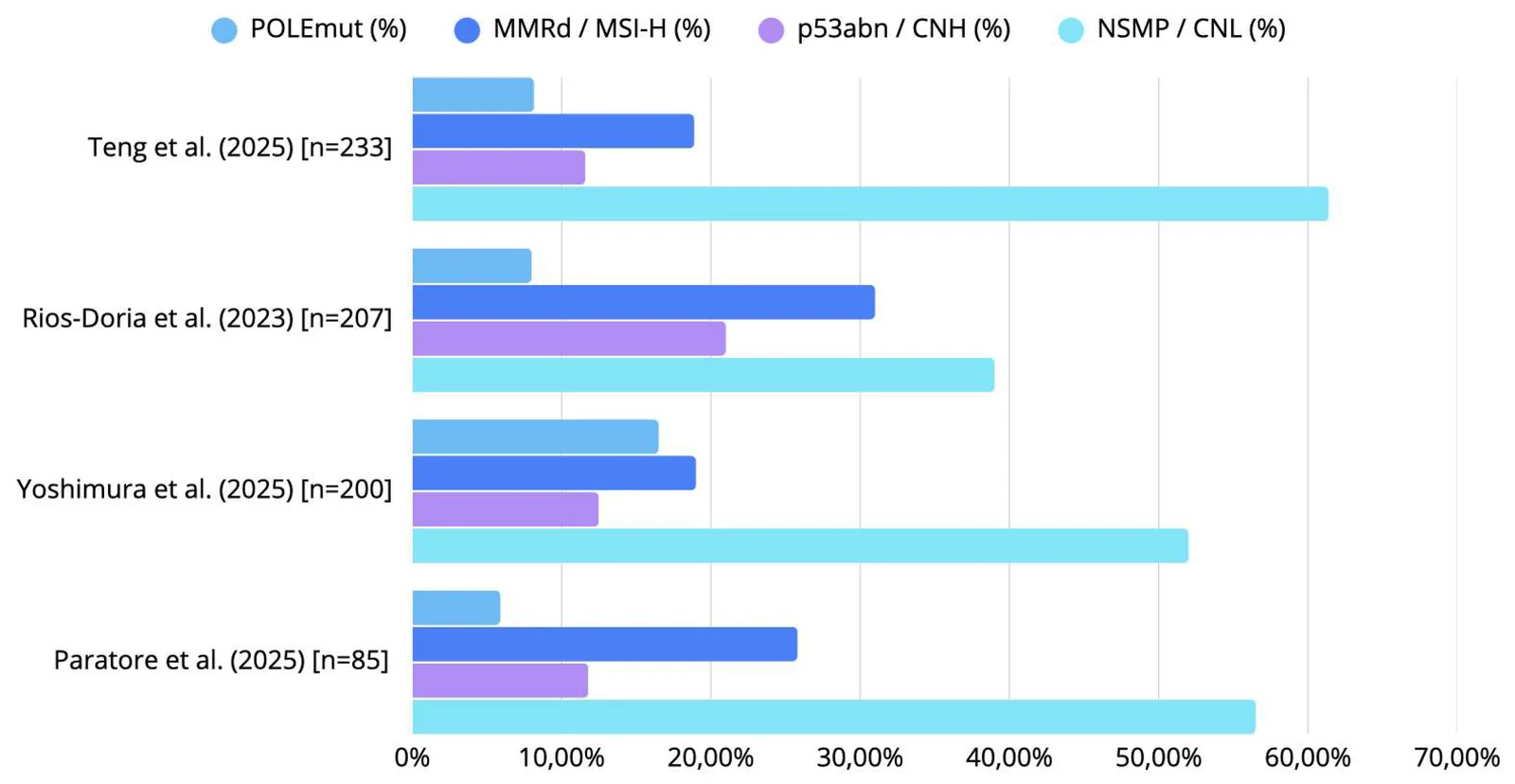
Comparison of the molecular subtype distribution of endometrial cancer across key genomic studies [31,32,33,35].

**Table 1:** Molecular subtypes of endometrial cancer according to The Cancer Genome Atlas (TCGA) classification [12,13].

Lp	Abbreviation for Molecular Subtype of Cancer	Full Expansion of the Abbreviation for a Molecular Subtype of Cancer	Frequency (%)
1.	POLEmut	Mutations in the exonuclease domain of the POLE gene	7–10%
2.	MMRd	Deficiency in the mismatch repair (MMR) system	25–30%
3.	NSMP(p53 wt)	No specific molecular profile (non-specific molecular type)	35–40%
4.	p53abn	Mutation in the TP53 gene	8–15%

**Table 2:** Comparison of the original TCGA molecular classification and its clinically applicable surrogate classifiers, ProMisE and TransPORTEC.

Feature	TCGA [12,14]	ProMisE [14,15,17]	TransPORTEC [16,18]
Subgroup nomenclature	POLE (ultramutated); MSI (hypermutated); copy-number low; copy-number high	POLE EDM; MMR-D; p53 wild-type; p53 abnormal	POLE proofreading-mutant; MSI; p53-mutant; no specific molecular profile (NSMP)
Methods used for subgroup assignment	Array-based and sequencing methodologies applied to a large series of endometrioid and serous ECs	Sequential decision tree: (i) IHC for MMR proteins, (ii) targeted sequencing of the *POLE* exonuclease domain, (iii) p53 IHC	p53 IHC, PCR-based MSI testing and Sanger sequencing of *POLE*, combined with a 13-gene hotspot mutation panel
MMR/MSI assessment	MSI assay as part of integrated genomic analysis	IHC for MLH1, MSH2, MSH6 and PMS2 (two-panel PMS2/MSH6 in the validation cohort); loss of staining defines MMR-D	Promega MSI analysis system v1.2; instability at ≥2 markers defines MSI; MMR IHC used for confirmation; MLH1 promoter methylation assessed by methylation-specific PCR
POLE assessment	Sequencing as part of integrated genomic analysis	Targeted primers covering exons 9–14, amplified on Fluidigm Access Arrays and sequenced on Illumina MiSeq (ultra-deep sequencing)	Sanger sequencing of exons 9 and 13, which together contain >90% of pathogenic exonuclease domain mutations
p53/TP53 assessment	TP53 mutation status as part of integrated genomic analysis	p53 IHC; abnormal defined as complete absence of staining (null pattern) or strong/diffuse staining in >70% of tumour cells	p53 IHC; positive if >50% of tumour cells show strong nuclear staining or discrete geographical patterns; indefinite cases sequenced for TP53 exons 5–8
Tissue requirement	Fresh-frozen material with special sample handling	FFPE material, including diagnostic biopsy and curettage specimens	FFPE material
Cohorts	373 endometrial carcinomas	Discovery *n* = 141; confirmation *n* = 319 (full cohort *n* = 460); population-based validation *n* = 452	116 high-risk ECs (TransPORTEC initiative); 834 classified early-stage endometrioid ECs from the PORTEC-1 and -2 trials
Reported subgroup distribution	≈7% POLE; 28% MSI-high; 39% CN-low; 26% CN-high	Validation cohort: 9.3% POLE; 28.1% MMR-D; 50.4% p53wt; 12.2% p53abn	High-risk series: 12% POLE; 16% MSI; 38% NSMP; 34% p53-mutant. PORTEC-1/-2: 6% POLE; 26% MSI; 59% NSMP; 9% p53-mutant
Tumours with more than one classifying feature	Not reported as a separate category	3.4% of the full cohort and 1.8% of the validation cohort; assigned according to the first feature identified in the decision tree	3% of the PORTEC-1/-2 series; classification of these cases stated to require additional analyses such as mutational load and copy-number status
Applicability to preoperative specimens	Not applicable with the original methodology	Demonstrated: κ 0.86 (*n* = 57) and κ 0.88 (*n* = 156) between diagnostic and hysterectomy specimens	Molecular alterations shown to be concordant between curettage and hysterectomy; however, LVSI and histopathological parameters of the integrated risk model cannot be assessed preoperatively
Reported clinical implication	Four subgroups associated with differences in progression-free survival	Independent prognostic information beyond established clinicopathological factors; improved discrimination when combined with ESMO risk groups; early identification of women requiring Lynch syndrome testing	Molecular subgroups define distinct prognostic subsets among clinicopathologically high-risk tumours; integration with clinicopathological factors reclassifies high-intermediate risk patients into favourable (50%), intermediate (35%), and unfavourable (15%) groups

**Table 3:** Comparison of the principal methods used for the molecular classification of endometrial cancer [5,10,28,29,30,31,32,33,34].

Criterion	Immunohistochemistry (IHC)	Targeted NGS Panel	Whole-Genome/Whole-exome Sequencing
What is detected	Protein expression: loss, reduced expression, or aberrant staining patterns	Sequence variants, small insertions/deletions, microsatellite instability, and, with appropriate panels, copy-number changes	The entire genome or coding sequence, including structural variants and copy-number profile
Detection of *POLE* exonuclease domain mutations	Not possible	Yes; the principal indication for sequencing in EC classification	Yes
Assessment of MMR/MSI status	Yes, by staining for MLH1, PMS2, MSH2, and MSH6; the preferred approach per current guidelines	Yes, by analysis of microsatellite loci; concordance with IHC reported between 91.8% and 97.6%	Yes
Assessment of p53/TP53 status	Yes, but atypical and subclonal patterns may be difficult to interpret; concordance with sequencing reported between 65.3% and 92.3%	Yes, by sequencing the coding region of *TP53*; large deletions and splice-site variants may be missed	Yes
Resolution of multiple classifiers	Not possible; tumours with more than one molecular feature cannot be correctly assigned without *POLE* status	Yes; this is the principal diagnostic advantage of sequencing in EC	Yes
Detection of additional actionable alterations	Limited to the markers stained	Yes; *PIK3CA*, *PTEN*, *KRAS*, *ERBB2*, *ESR1,* and homologous recombination repair genes are covered by standard panels	Yes, most comprehensively
Material required	Small tissue sample; applicable to biopsy and curettage specimens	FFPE-derived DNA; applicable to biopsy specimens, with a minimum tumour cell content of approximately 30%	Larger amount of good-quality DNA
Turnaround time	Approximately 1–2 days	Approximately 5–10 days	Longest, owing to the volume of data generated
Cost and infrastructure	Low; routinely available in pathology laboratories	Higher; requires dedicated equipment, bioinformatic infrastructure, and experienced personnel	Highest; the most computationally intensive approach
Role in EC classification	Sufficient for the identification of MMRd and p53abn; the basis of surrogate classifiers	Required for *POLE* status and for tumours with more than one molecular feature; enables single-test classification	Research tool; not used for routine classification
Clinical relevance for molecular classification of EC	It enables the direct determination of MMRd and p53abn status, which leads to the patient being referred for testing for Lynch syndrome and the identification of candidates for therapy based on immune checkpoint inhibitors (MMRd) or, where appropriate, assessment using PARP inhibitors (p53abn/HRD phenotype); it does not allow for the identification of POLE mutations, so POLE-driven tumours may be incorrectly classified as high-risk and subjected to overtreatment unless sequencing is carried out.	It confirms POLE status, which is a key factor in selecting candidates for reduced-intensity adjuvant therapy, and resolves cases where tumours exhibit more than one classification feature by prioritising POLE over p53abn; it detects genetic alterations associated with a high risk of disease recurrence (HRR) (BRCA1/2, ATM, CHEK2), which are relevant for eligibility for treatment with PARP inhibitors, and alterations enabling specific therapeutic interventions (PIK3CA, PTEN, KRAS, ERBB2, ESR1), which allow for a more precise selection of treatment within the NSMP subgroup.	It provides the most comprehensive molecular profile underpinning the original TCGA subgroups; however, due to the costs, the time taken to obtain results, and the complexity of the test, it is not used to inform individual treatment decisions in routine practice. Its clinical application in the classification of endometrial cancer remains at the research stage.

**Table 4:** Characteristics of the 11 included studies.

Study (Author, Year)	Study Design	Sample Size	Appraisal Tool
Teng et al. (2025) [35]	Retrospective cohort	*n* = 233 (OS data for *n* = 131; median follow-up 66 months)	NOS
Guo et al., ESMO Open (2024) [28]	Retrospective study on a consecutive cohort (NGS vs. IHC)	*n* = 331 (284 with endometrioid carcinoma)	QUADAS-2
Jamieson et al. (2023) [29]	Retrospective validation of a single-test NGS classifier against the original multi-test ProMisE algorithm	*n* = 164 (incl. 15 matched biopsy–hysterectomy pairs)	QUADAS-2
Dedeurwaerdere et al. (2021) [30]	Comparative diagnostic-accuracy study	*n* = 49 tumour samples (colorectal and endometrial cancer)	QUADAS-2
Guo et al., Front. Genet. (2024) [9]	Retrospective cohort	*n* = 90 (FFPE tissue and blood; treated Mar 2022–Dec 2023)	NOS
Rios-Doria et al. (2023) [31]	Retrospective cohort	*n* = 1834 (preoperative biopsy or hysterectomy specimens)	NOS
Rivera et al. (2023) [36]	Retrospective, non-consecutive/matched cohort (high-grade endometrioid carcinomas, 2018–2020)	*n* = 63 (60 analysed)	QUADAS-2
Yoshimura et al. (2025) [32]	Retrospective cohort	*n* = 200 (surgery incl. hysterectomy)	QUADAS-2
Paratore et al. (2025) [33]	Observational cohort study	*n* = 85 (tumour tissue)	QUADAS-2
Valtcheva et al. (2017) [37]	Case report	*n* = 1 (synchronous endometrioid carcinoma)	Not applicable
Alba Mota et al. (2022) [38]	Translational multi-region sequencing study	*n* = 9 patients (42 primary tumour regions, 30 metastatic samples)	NOS

**Table 5:** Variables collected using the standardised data extraction form.

Domain	Variables Extracted
Study characteristics	First author, year of publication, country, study design, sample size
Patient and tumour characteristics	Histological subtype, FIGO stage, grade, clinical context
Molecular classification methods	NGS platform, gene panel used, IHC-based surrogate classifiers, other molecular techniques
TCGA molecular subgroups assessed	POLE mutation; MSI-H/MMRd; CNL/NSMP; CNH/p53abn
Outcomes of interest	Concordance between NGS- and IHC-based classification; reclassification rates; diagnostic ambiguities resolved by NGS; prognostic associations; therapeutic implications; identification of actionable mutations
Technical parameters	Tumour cell content requirements, turnaround time, cost
Reported limitations	Bioinformatic complexity, cost, tissue requirements, interpretative difficulties

**Table 6:** Comparison of two studies: Yoshimura et al. (2025) and Paratore et al. (2025) [32,33].

Feature of NGS Utility	Yoshimura et al. (2025) [32]	Paratore et al. (2025) [33]
Primary NGS Advantage	NGS-based classification provides more precise OS (overall survival) stratification than IHC-based methods.	NGS identifies specific molecular targets (e.g., *BRCA1/2*, *PIK3CA*) for personalised medicine.
Genomic Resolution	Capable of detecting non-canonical and subclonal mutations that escape standard IHC screening.	High diagnostic yield in identifying actionable mutations for PARP inhibitors and immunotherapy.
Handling Misclassification	Specifically addresses the “multiple classifier” problem, correctly reassigning cases with overlapping IHC patterns.	Focuses on refining the “NSMP” (No Specific Molecular Profile) group into more distinct prognostic categories.
Diagnostic Accuracy	Proves that NGS reduces the “grey zone” of ambiguous p53 and MMR results compared to the ProMisE algorithm.	Demonstrates 100% feasibility in integrating NGS into the standard clinical workflow for high-grade cases.
Data Interpretation	Uses high-throughput sequencing to establish a more objective, digital-based molecular signature.	Emphasises the role of NGS in guiding adjuvant treatment (e.g., deciding between observation and chemotherapy).
Specimen Efficiency	Highlights the ability to obtain a full molecular profile even from small, fragmented biopsy samples.	Showcases NGS as a cost-effective and tissue-saving alternative to multiple separate IHC stains.

## Data Availability

The original contributions presented in this study are included in the article/Supplementary Materials. Further inquiries can be directed to the corresponding author.

## Supplementary Materials

The following supporting information can be downloaded at: https://www.mdpi.com/article/10.3390/ijms27198602/s1. References [9,28,29,30,31,32,33,35,36,37,38,62] are cited in the Supplementary Materials.

## Abbreviations

The following abbreviations are used in this manuscript:

EC	Endometrial cancer
TCGA	The Cancer Genome Atlas
POLEmut	Mutations in the exonuclease domain of the POLE gene
MMRd	Deficiency in the mismatch repair system
MMR	Mismatch repair
NSMP	No specific molecular profile
p53 wt	p53 wild-type
p53abn	Mutation in the TP53 gene (p53 abnormal)
PRISMA	Preferred Reporting Items for Systematic Reviews and Meta-Analyses
NGS	Next-generation sequencing
IHC	Immunohistochemistry
ProMisE	Proactive Molecular Risk Classifier for Endometrial Cancer
TransPORTEC	Translational Portec (consortium/classifier)
FIGO	International Federation of Gynecology and Obstetrics
MSI-H	High microsatellite instability
CNL	Low copy number
CNH	High copy number
NOS	Newcastle–Ottawa Scale
QUADAS-2	Quality Assessment of Diagnostic Accuracy Studies-2
AGREE II	Appraisal of Guidelines for Research and Evaluation II
AMSTAR 2	Assessment of Multiple Systematic Reviews 2
PCR	Polymerase chain reaction
RNA-seq	RNA sequencing
ChIP-seq	Chromatin immunoprecipitation sequencing
WGS	Whole-genome sequencing
MSI	Microsatellite stable
FFPE	Formalin-fixed paraffin-embedded
OS	Overall survival
DSS	Disease-specific survival
cDNA	Complementary DNA
TME	Tumour microenvironment
mIF	Multiplex immunofluorescence
mIHC	Multiplex immunohistochemistry
MSH-6	MutS homologue 6
PMS-2	Postmeiotic segregation increased 2
MLH-1	MutL homolog 1
MSH-2	MutS homologue 2
HRR	Homologous recombination repair
LVSI	Lymphovascular space invasion
AUC	Area under the curve
TAT	Turnaround time
LS	Lynch syndrome
HRD	Homologous recombination deficiency
LOH	Loss of heterozygosity
PARP	Poly (ADP-ribose) polymerase
EBRT	External beam radiotherapy
ISGyP	International Society of Gynecological Pathology
