# Supplementary material for: Refining the TCGA Framework: The Pivotal Role of Targeted Next-Generation Sequencing in Resolving Diagnostic Ambiguities in Endometrial Cancer—Systematic Review
Source: Int J Mol Sci. 2026 Sep 25;27(19):8602. doi: 10.3390/ijms27198602 (PMC13665446; doi:10.3390/ijms27198602)
Supplement: Supplementary file 1 [file ijms-27-08602-s001.zip › ijms-4548612-supplementary-PRISMA Checklist.pdf]

PRISMA 2020 Checklist

Section and Topic	Item #	Checklist item	Location where item is reported
**TITLE**			
Title	1	Identify the report as a systematic review.	Title page. Manuscript title identifies it as a systematic review.
**ABSTRACT**			
Abstract	2	See the PRISMA 2020 for Abstracts checklist.	Abstract (Background/Methods/Results/Conclusions).
**INTRODUCTION**			
Rationale	3	Describe the rationale for the review in the context of existing knowledge.	Section 1. Introduction.
Objectives	4	Provide an explicit statement of the objective(s) or question(s) the review addresses.	Stated in the Abstract only; not restated explicitly in Section 1. Introduction.
**METHODS**			
Eligibility criteria	5	Specify the inclusion and exclusion criteria for the review and how studies were grouped for the syntheses.	Section 4. Materials and Methods — "Eligibility Criteria."
Information sources	6	Specify all databases, registers, websites, organisations, reference lists and other sources searched or consulted to identify studies. Specify the date when each source was last searched or consulted.	Section 4 — "Search Strategy" (PubMed, Scopus, Embase). Gives search start date (Oct 2025) only, not a last-searched date; no registers/grey literature noted.
Search strategy	7	Present the full search strategies for all databases, registers and websites, including any filters and limits used.	Section 4 — "Search Strategy" (full Boolean strings and per-database hit counts given).
Selection process	8	Specify the methods used to decide whether a study met the inclusion criteria of the review, including how many reviewers screened each record and each report retrieved, whether they worked independently, and if applicable, details of automation tools used in the process.	Section 4 — "Study Selection" (5 reviewers independently; 6 further reviewers adjudicated disagreements).
Data collection process	9	Specify the methods used to collect data from reports, including how many reviewers collected data from each report, whether they worked independently, any processes for obtaining or confirming data from study investigators, and if applicable, details of automation tools used in the process.	Section 4 — "Data Extraction" (5 reviewers, standard form; discrepancies to 6th/7th reviewer).
Data items	10a	List and define all outcomes for which data were sought. Specify whether all results that were compatible with each outcome domain in each study were sought (e.g. for all measures, time points, analyses), and if not, the methods used to decide which results to collect.	Section 4 — "Data Extraction" (concordance/reclassification rates, prognostic and therapeutic implications).
	10b	List and define all other variables for which data were sought (e.g. participant and intervention characteristics, funding sources). Describe any assumptions made about any missing or unclear information.	Section 4 — "Data Extraction" (study/patient/tumour characteristics, NGS platforms/panels, technical parameters).
Study risk of bias assessment	11	Specify the methods used to assess risk of bias in the included studies, including details of the tool(s) used, how many reviewers assessed each study and whether they worked independently, and if applicable, details of automation tools used in the process.	Section 4 — "Quality Assessment" (NOS, QUADAS-2, AGREE II, AMSTAR 2 by study type; 2 reviewers).
Effect measures	12	Specify for each outcome the effect measure(s) (e.g. risk ratio, mean difference) used in the synthesis or presentation of results.	Not applicable — descriptive/narrative synthesis used, no pooled effect measure (see item 13d).
Synthesis methods	13a	Describe the processes used to decide which studies were eligible for each synthesis (e.g. tabulating the study intervention characteristics and comparing against the planned groups for each synthesis (item #5)).	Section 4 — "Data Synthesis and Analysis" (7 thematic areas defined for the narrative synthesis).

Section and Topic	Item #	Checklist item	Location where item is reported
	13b	Describe any methods required to prepare the data for presentation or synthesis, such as handling of missing summary statistics, or data conversions.	Not reported — no method described for handling missing data or data conversions.
	13c	Describe any methods used to tabulate or visually display results of individual studies and syntheses.	Tables 1, 2, 4, 5 and Figures 1–2. Note: text also cites a "Table 3" of included studies (Sec. 4, Study Selection) that does not exist in the manuscript — see item 17 (Results, #17) below.
	13d	Describe any methods used to synthesize results and provide a rationale for the choice(s). If meta-analysis was performed, describe the model(s), method(s) to identify the presence and extent of statistical heterogeneity, and software package(s) used.	Narrative synthesis; explicitly no meta-analysis ("heterogeneity of research projects and molecular platforms"), Section 4.
	13e	Describe any methods used to explore possible causes of heterogeneity among study results (e.g. subgroup analysis, meta-regression).	Not applicable — no meta-analysis conducted.
	13f	Describe any sensitivity analyses conducted to assess robustness of the synthesized results.	Not applicable — no meta-analysis conducted.
Reporting bias assessment	14	Describe any methods used to assess risk of bias due to missing results in a synthesis (arising from reporting biases).	Not reported — no method for assessing reporting/publication bias described.
Certainty assessment	15	Describe any methods used to assess certainty (or confidence) in the body of evidence for an outcome.	Not reported — no certainty-of-evidence (e.g. GRADE) method described.
**RESULTS**			
Study selection	16a	Describe the results of the search and selection process, from the number of records identified in the search to the number of studies included in the review, ideally using a flow diagram.	Section 4 — "Study Selection" and Figure 1 (PRISMA flow diagram): 13,669 records → 403 screened → 62 full texts → 14 included. (Reported within Materials and Methods rather than Results, per journal template order.)
	16b	Cite studies that might appear to meet the inclusion criteria, but which were excluded, and explain why they were excluded.	Section 4 — "Study Selection" gives aggregate exclusion counts by reason at full-text stage; individual excluded studies are not cited by name.
Study characteristics	17	Cite each included study and present its characteristics.	Referenced as "Table 3" (Sec. 4, Study Selection) but no such table exists in the manuscript — the 14 included studies are otherwise described only in narrative form (Section 2). Recommend reinstating a study-characteristics table.
Risk of bias in studies	18	Present assessments of risk of bias for each included study.	Not reported — quality-assessment tools are described (Section 4) but no per-study risk-of-bias results are presented anywhere.
Results of individual studies	19	For all outcomes, present, for each study: (a) summary statistics for each group (where appropriate) and (b) an effect estimate and its precision (e.g. confidence/credible interval), ideally using structured tables or plots.	Section 2. Results and 2.1 (narrative findings per study, e.g. Qiuli Teng et al. 2025, Rios-Doria et al. 2023, Yoshimura et al. 2025, Paratore et al. 2025), plus Tables 4–5. Given as point estimates without confidence intervals.
Results of syntheses	20a	For each synthesis, briefly summarise the characteristics and risk of bias among contributing studies.	Section 2. Results (introductory synthesis) and Section 4 — "Data Synthesis and Analysis." Risk of bias among contributing studies is not summarised (see item 18, Results).
	20b	Present results of all statistical syntheses conducted. If meta-analysis was done, present for each the summary estimate and its precision (e.g. confidence/credible interval) and measures of statistical heterogeneity. If comparing groups, describe the direction of the effect.	Not applicable — no statistical synthesis/meta-analysis performed.
	20c	Present results of all investigations of possible causes of heterogeneity	Not applicable — no heterogeneity investigation performed.

PRISMA 2020 Checklist

Section and Topic	Item #	Checklist item	Location where item is reported
		among study results.	
	20d	Present results of all sensitivity analyses conducted to assess the robustness of the synthesized results.	Not applicable — no sensitivity analyses performed.
Reporting biases	21	Present assessments of risk of bias due to missing results (arising from reporting biases) for each synthesis assessed.	Not reported.
Certainty of evidence	22	Present assessments of certainty (or confidence) in the body of evidence for each outcome assessed.	Not reported — no certainty (e.g. GRADE) ratings presented for any outcome.
**DISCUSSION**			
Discussion	23a	Provide a general interpretation of the results in the context of other evidence.	Section 3. Discussion.
	23b	Discuss any limitations of the evidence included in the review.	Not clearly addressed — Section 3.1 discusses limitations of NGS as a technology, not limitations of the included evidence base (e.g. study heterogeneity, risk of bias, small samples).
	23c	Discuss any limitations of the review processes used.	Not reported — limitations of the review process itself (3 databases only, English-language restriction, no meta-analysis, possible missed literature) are not discussed.
	23d	Discuss implications of the results for practice, policy, and future research.	Partially addressed — Section 5. Conclusions covers practice implications; explicit future-research recommendations are not stated.
**OTHER INFORMATION**			
Registration and protocol	24a	Provide registration information for the review, including register name and registration number, or state that the review was not registered.	Section 4 — "Study Design": registered in PROSPERO, registration number CRD420261356093.
	24b	Indicate where the review protocol can be accessed, or state that a protocol was not prepared.	Section 4 — "Study Design" gives the registration number only; no separate protocol access link is stated.
	24c	Describe and explain any amendments to information provided at registration or in the protocol.	Not applicable — no amendments reported.
Support	25	Describe sources of financial or non-financial support for the review, and the role of the funders or sponsors in the review.	Funding statement (end matter): no external funding.
Competing interests	26	Declare any competing interests of review authors.	Conflicts of Interest statement (end matter): none declared.
Availability of data, code and other materials	27	Report which of the following are publicly available and where they can be found: template data collection forms; data extracted from included studies; data used for all analyses; analytic code; any other materials used in the review.	Data Availability Statement (end matter): no new data; available within the article and cited references.

From: Page MJ, McKenzie JE, Bossuyt PM, Boutron I, Hoffmann TC, Mulrow CD, et al. The PRISMA 2020 statement: an updated guideline for reporting systematic reviews. BMJ 2021;372:n71. doi: 10.1136/bmj.n71. This work is licensed under CC BY 4.0. To view a copy of this license, visit https://creativecommons.org/licenses/by/4.0/ [62]
