# Supplementary material for: Refining the TCGA Framework: The Pivotal Role of Targeted Next-Generation Sequencing in Resolving Diagnostic Ambiguities in Endometrial Cancer—Systematic Review
Source: Int J Mol Sci. 2026 Sep 25;27(19):8602. doi: 10.3390/ijms27198602 (PMC13665446; doi:10.3390/ijms27198602)
Supplement: Supplementary file 1 [file ijms-27-08602-s001.zip › IJMS-4548612-Supplementary_table_S1_NGS_updated.-9.25docx.pdf]

Supplementary Table S1. Characteristics and main findings of the studies included in the review.

All eleven studies providing original data on NGS-based molecular classification of endometrial cancer, corresponding to the studies listed in Table 3 and appraised in the Quality Assessment (Section 2) .

Study (full citation)	Study design	Cohort and specimen	Sequencing methodology	Classification approach / system	Main findings	Relevance to review objectives
Teng, Q.; Yuan, Z.; Mu, Y.; Ma, X.; Wang, S.; Sun, C.; Chin, L.; Huang, Z.; Zhu, C.; Yin, A.; et al. Molecular Subtyping of Endometrial Cancer via a Simplified One-Step NGS Classifier, ARID1A and ZFHX4 Mutations Help Further Subclassify CNL/MSI-H Patients. Diagn. Pathol. 2025, 20 (1), 52. https://doi.org/10.1186/s13000-025-01652-z [35]	Retrospective cohort	233 FFPE samples; overall survival data available for 131 patients; median follow-up 66 months	Simplified one-step NGS panel covering POLE, TP53 and microsatellite instability	TCGA-based, assigned by NGS alone	POLE 8.15%, MSI-H 18.88%, CNH 11.59%, CNL 61.37%. Ten-year overall and disease-specific survival 100% in the POLE subtype versus 33.51% and 39.69% in CNH.	Demonstrates that a simplified single-step NGS classifier stratifies patients into four prognostic molecular subtypes.
Guo, Q.; Tang, S.; Ju, X.; Feng, Z.; Zhang, Z.; Peng, D.; Liu, F.; Du, H.; Wang, J.; Zhang, Y.; et al. Identification of Molecular Subtypes for Endometrial Carcinoma Using a 46-Gene Next-Generation Sequencing Panel: A Retrospective Study on a Consecutive Cohort. ESMO Open 2024, 9 (10), 103710. https://doi.org/10.1016/j.esmoop.2024.103710 [28]	Retrospective study on a consecutive cohort	331 patients, of whom 284 had endometrioid carcinoma	46-gene targeted NGS panel	TCGA surrogate; NGS compared with IHC	Agreement with IHC 91.8% for MSI-H/MMR status and 65.3% for p53/TP53 status. Pathogenic homologous recombination repair variants: BRCA1 3.6%, BRCA2 9.7%, ATM 12.7%, CHEK2 2.7%.	Quantifies discordance between IHC and NGS and identifies potentially actionable homologous recombination repair alterations.
Jamieson, A.; McConechy, M. K.; Lum, A.; Leung, S.; Thompson, E. F.; Senz, J.; Talhouk, A.; Huntsman, D. G.; Bashashati, A.; Gilks, C. B.; et al. Harmonized Molecular Classification; Assessment of a Single-Test ProMisE NGS Tool. Gynecol. Oncol. 2023, 175, 45–52. https://doi.org/10.1016/j.ygyno.2023.05.073 [29]	Retrospective validation of a single-test classifier	164 endometrial carcinomas previously classified by ProMisE, including 15 matched biopsy and hysterectomy pairs; FFPE	Amplicon-based NGS panel (37 genes, 21 microsatellite loci) sequenced on Illumina MiSeq; variant allele frequency threshold 5%	ProMisE-NGS compared with the original multi-test ProMisE	159 of 164 cases concordant (97%), kappa 0.96, accuracy 0.97. All 15 biopsy–hysterectomy pairs concordant. Prognostic separation of the four subtypes preserved.	Shows that a single DNA-based NGS test can replace the multi-platform surrogate algorithm without loss of prognostic value.
Guo, Y.; Yan, G.; Zhang, P.; Liu, Y.; Zhao, C.; Zeng, X. The Clinical Utility of Next Generation Sequencing in Endometrial Cancer: Focusing on Molecular Subtyping and Lynch Syndrome. Front. Genet. 2024, 15. https://doi.org/10.3389/fgene.2024.1440971 [9]	Retrospective cohort	90 patients treated between March 2022 and December 2023; FFPE tumour tissue and peripheral blood	Illumina NGS covering POLE exons 3–14 and part of exon 19, the entire TP53 coding region and 55 microsatellite loci; separate germline MMR gene panel	TCGA-based, assigned by NGS; combined with germline screening for Lynch syndrome	POLE 12.2%, MMRd 21.1%, p53abn 6.7%, NSMP 60%. Pathogenic germline MMR variants in 9 of 90 patients (10%), of whom 7 were MMRd and 2 were NSMP.	Demonstrates that molecular subtype alone does not substitute for germline testing and that IHC-based algorithms may miss Lynch syndrome cases.
Dedeurwaerdere, F.; Claes, K. B.; Van Dorpe, J.; Rottiers, I.; Van der Meulen, J.; Breyne, J.; Swaerts, K.; Martens, G. Comparison of Microsatellite Instability Detection by Immunohistochemistry and Molecular Techniques in Colorectal and Endometrial Cancer. Sci. Rep. 2021, 11 (1), 12880. https://doi.org/10.1038/s41598-021-91974-x [30]	Comparative diagnostic accuracy study	49 tumour samples from colorectal and endometrial cancer; FFPE	NGS-based microsatellite instability detection	NGS compared with MMR IHC, PCR-based MSI testing and the Idylla platform	Area under the curve 0.93 for NGS versus 0.91 for both PCR and Idylla. All three molecular tests reached 100% sensitivity and specificity when tumour cell content was at least 30%.	Quantifies agreement between molecular and immunohistochemical MMR assessment and defines the tumour-content threshold required.

Study (full citation)	Study design	Cohort and specimen	Sequencing methodology	Classification approach / system	Main findings	Relevance to review objectives
Rios-Doria, E.; Momeni-Boroujeni, A.; Friedman, C. F.; Selenica, P.; Zhou, Q.; Wu, M.; Marra, A.; Leitao, M. M.; Iasonos, A.; Alektiar, K. M.; et al. Integration of Clinical Sequencing and Immunohistochemistry for the Molecular Classification of Endometrial Carcinoma. Gynecol. Oncol. 2023, 174, 262–272. https://doi.org/10.1016/j.ygyno.2023.05.059 [31]	Retrospective cohort	1,834 patients; preoperative biopsy or hysterectomy specimens	Clinical tumour-normal targeted sequencing	Integrated NGS and IHC algorithm; TCGA	NSMP 31.3%, MMRd 23.3%, p53abn 40%, POLEmut 5.3%. Concordance between IHC and NGS exceeded 95%. 6% of cases showed more than one molecular feature.	Largest cohort supporting an integrated algorithm in which sequencing is reserved for POLE status and for ambiguous cases.
Yoshimura, T.; Nakamura, K.; Chiyoda, T.; Takamatsu, R.; Kawano, R.; Aimono, E.; Kawaida, M.; Sakai, K.; Masugi, Y.; Nishihara, H.; et al. Next-Generation Sequencing Outperforms Proactive Molecular Risk Classifier for Endometrial Cancer (ProMisE) in Endometrial Cancer Molecular Classification. BJC Rep. 2025, 3 (1), 37. https://doi.org/10.1038/s44276-025-00145-2 [32]	Retrospective cohort	200 patients who underwent surgery including hysterectomy	Targeted NGS panel	NGS-based classification compared with the IHC-based ProMisE classifier	CN-L 52.0%, MSI-H 19.0%, POLE 16.5%, CN-H 12.5%. Overall survival differed significantly across NGS-defined subtypes but not across ProMisE groups. 18.2% of POLE cases showed concurrent MSI-H; 48% of CN-H cases showed a wild-type p53 pattern on IHC.	Provides head-to-head evidence of superior prognostic discrimination of NGS over the immunohistochemical surrogate.
Rivera, D.; Paudice, M.; Accorsi, G.; Valentino, F.; Ingaliso, M.; Pianezzi, A.; Roggieri, P.; Trevisan, L.; Buzzatti, G.; Mammoliti, S.; et al. The Advantages of Next-Generation Sequencing Molecular Classification in Endometrial Cancer Diagnosis. J. Clin. Med. 2023, 12 (23), 7236. https://doi.org/10.3390/jcm12237236 [36]	Retrospective study	63 high-grade endometrioid carcinomas operated between 2018 and 2020; hysterectomy specimens	Targeted NGS with Sanger sequencing for confirmation	NGS compared with p53 IHC; TCGA	Of 20 TP53-mutated cases, two were discordant: EC-50 (Gly266Arg, detected only on repeat testing of better-quality material) and EC-58 (Gly245Asp with normal p53 expression).	Shows that immunohistochemistry alone is insufficient for definitive subtyping in borderline and high-grade cases.
Paratore, S.; Russo, A.; Blanco, G.; Lanzafame, K.; Giurato, E.; Bartoloni, G.; D'Asta, M.; Sapienza, M.; Solarino, V.; Vinci, V.; et al. Clinical Application of Next-Generation Sequencing for Molecular Classification in the Management of Endometrial Cancer: An Observational Cohort Study. Cancers 2025, 17 (11), 1806. https://doi.org/10.3390/cancers17111806 [33]	Observational cohort study	85 patients; tumour tissue	Multigene targeted NGS panel	NGS compared with IHC; TCGA	POLE 5.9%, MMRd/MSI 25.8%, p53abn 11.8%, NSMP 56.5%. Concordance 97.6% for MSI/MMR and 92.3% for p53/TP53. Actionable PIK3CA, PTEN, KRAS, ERBB2 and ESR1 alterations identified.	Demonstrates feasibility of NGS in a routine clinical workflow and its role in identifying targets for personalised therapy.
Valtcheva, N.; Lang, F. M.; Noske, A.; Samartzis, E. P.; Schmidt, A.-M.; Bellini, E.; Fink, D.; Moch, H.; Rechsteiner, M.; Dedes, K. J.; et al. Tracking the Origin of Simultaneous Endometrial and Ovarian Cancer by Next-Generation Sequencing – a Case Report. BMC Cancer 2017, 17 (1), 66. https://doi.org/10.1186/s12885-017-3054-6 [37]	Case report	One patient with synchronous endometrioid carcinoma of the corpus uteri and left ovary, with lung metastasis four years later	Targeted NGS panel covering 409 cancer-associated genes	Not applicable; used for clonality assessment	Mutation profiles of the endometrial and ovarian tumours were 67% identical, indicating shared origin, whereas the lung tumour showed a distinct profile.	Illustrates the use of NGS for differential diagnosis where the distinction between synchronous primaries and metastasis alters staging and therapeutic strategy.
Mota, A.; Oltra, S. S.; Selenica, P.; Moiola, C. P.; Casas-Arozamena, C.; López-Gil, C.; Diaz, E.; Gatius, S.; Ruiz-Miro, M.; Calvo, A.; et al.	Translational multi-region	9 metastatic endometrial cancer patients; 42 primary	Whole-exome sequencing at median 52x depth with AmpliSeq targeted	ProMisE surrogate markers used for	532 somatic variants validated. Intratumour genetic heterogeneity highest in MSI tumours and lowest in	Demonstrates that NGS can reconstruct clonal evolution and identify actionable

Study (full citation)	Study design	Cohort and specimen	Sequencing methodology	Classification approach / system	Main findings	Relevance to review objectives
Intratumor Genetic Heterogeneity and Clonal Evolution to Decode Endometrial Cancer Progression. Oncogene 2022, 41 (13), 1835–1850. https://doi.org/10.1038/s41388-022-02221-0 [38]	sequencing study	tumour regions and 30 metastatic samples; fresh-frozen and FFPE tissue, one uterine aspirate and five patient-derived xenografts	validation; array comparative genomic hybridisation for serous cases	TCGA subtype assignment	copy-number low tumours. An ATM splice-site mutation in an ambiguous carcinoma predicted response to olaparib, confirmed in xenograft models.	targets in tumours that do not fit the standard subtypes.

Abbreviations: CN-H/CNH, copy-number high; CN-L/CNL, copy-number low; EC, endometrial cancer; FFPE, formalin-fixed paraffin-embedded; IHC, immunohistochemistry; MMR, mismatch repair; MMRd, mismatch repair deficient; MSI, microsatellite instability; MSI-H, high microsatellite instability; NGS, next-generation sequencing; NSMP, no specific molecular profile; p53abn, p53 abnormal; POLEmut, POLE-mutated; ProMisE, Proactive Molecular Risk Classifier for Endometrial Cancer; TCGA, The Cancer Genome Atlas.
